# Microbe-driven flavonoid biotransformation revealed by multi-omics analysis during Hua Feng Dan Yao Mu fermentation

**DOI:** 10.3389/fmicb.2026.1816282

**Published:** 2026-06-17

**Authors:** Qilin Shu, Xue Zhang, Lingling Liu, Yayang Gao, Yan Yang, Youli Chen, Yinhao Liao, Jian Xu, Yongping Zhang, Ling Guo, Guoqiong Cao

**Affiliations:** 1 College of Pharmaceutical Sciences, Guizhou University of Traditional Chinese Medicine, Guiyang, China; 2 Zun Yi Liaoyuanhetang Pharmaceutical Co., Ltd., Zunyi, China; 3 Guizhou Engineering Technology Research Center for Processing and Preparation of Traditional Chinese Medicine and Ethnic Medicine, Guizhou University of Traditional Chinese Medicine, Guiyang, China; 4 National Engineering Technology Research Center for Miao Medicine, Guizhou University of Traditional Chinese Medicine, Guiyang, China; 5 Guizhou Key Laboratory for Germplasm Innovation and Resource-Efficient Utilization of Dao-di Herbs, Guiyang, China

**Keywords:** *Aspergillus niger*, *Bacillus amyloliquefaciens*, flavonoid transformation, Hua Feng Dan Yao Mu, microbial succession

## Abstract

Microbes play an important role in the transformation of bioactive components in traditional Chinese medicine (TCM) fermentation. It is not understood how the microbial succession relates to the changes in active components of Hua Feng Dan Yao Mu (HFDYM) fermentation. This study systematically characterized the chemical and microbial dynamics during HFDYM fermentation, using multi-omics analyses, with a focus on flavonoid transformation. Single-strain fermentation identified specific microorganisms involved in flavonoid metabolism. During fermentation, there were significant abundance changes in 60 flavonoids. Metabolic pathway analysis revealed a distinct remodeling of flavonoid metabolism, marked by increased levels of (+)-catechin and naringin alongside decreases in many other flavonoids. The analysis of the microbial community showed that the dominant bacterial genera were *Tetragenococcus*, *Levilactobacillus*, and *Bacillus* while the dominant fungi were *Wickerhamomyces* and *Aspergillus*. Further correlation studies related the *Bacillus* and *Aspergillus* to 10 flavonoids. A follow-up fermentation using *Bacillus amyloliquefaciens* and *Aspergillus niger* enhanced the total flavonoid content significantly, which effectively promotes the accumulation of (+)-catechin, luteolin and kaempferol. Moreover, it accelerated the degradation of vitexin and eriodictyol. The findings elucidate the mechanism of microbial-mediated flavonoid changes occurred in HFDYM fermentation. This would provide a scientific basis for its process standardization and optimization to improve TCM quality.

## Graphical abstract

**Figure:**
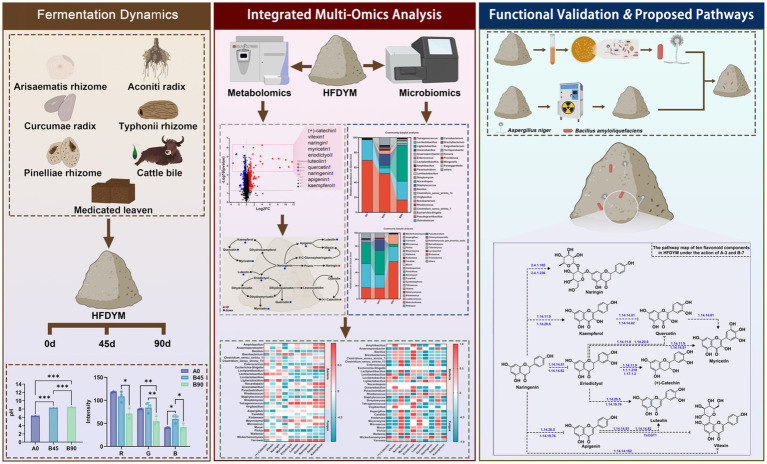

## 1 Introduction

Solid-state fermentation (SSF) is a well-known microbial technique used in food production (Krishna, 2005; Canoy et al., 2024), alcoholic beverage processing (Fang et al., 2019) and TCM (Liu et al., 2022). In TCM preparation, bacteria are grown directly on solid herbal substrates under low humidity to optimize their pharmacological profiles. As a well-known processing technique, herbal fermentation is able to improve the active components, improve therapeutic properties and reduce possible side effects (Wang et al., 2018; Zhang et al., 2023). Recent studies show significant benefits for such processes, such as fermenting *Salvia miltiorrhiza* with *Aspergillus oryzae* increases total phenolic and flavonoid yields by 1.2–1.3 fold and increases antioxidant activity (Moon and Cha, 2020); treating *Pueraria lobata* with *Limosilactobacillus fermentum* NCU001464 similarly increased flavonoid content enhancing antioxidant and xanthine oxidase inhibitory activity (Huang et al., 2025). Fermentation of *Polygonum multiflorum* with *Ganoderma lucidum* reduces stilbene glycoside level and reduces hepatotoxicity (Duan and Lin, 2023); bile fermented *Arisaema erubescens* had improved anticonvulsant properties, gained additional antipyretic effects, and significantly reduced death rates in mice (Shan et al., 2021). All these cases show that herbal fermentation is a promising and innovative approach to modernization and global integration of TCM.

The pharmaceutical effects of herbal fermentation arise from precise decomposition and directed conversion of organic molecules in herbs by functional microbes. By utilizing their unique enzymes and metabolic networks, functional microbes modify structure and chemically reconstruct natural products, precisely controlling their bioactivity (Song et al., 2024; Zhang et al., 2024). Studies show that strain specificity and substrate dependence vary considerably. For example, fermenting Lian-Zhi-Fan solution with *Penicillium expansum* and *Aspergillus niger* promotes deglycosylation of iridoid glycosides and reduces fermentation time (Xie et al., 2021), while fermenting kombucha with multi-strain consortium significantly alters composition and content of various components, including total flavonoids, polyphenols and catechins (Liang et al., 2024; Sknepnek et al., 2021). However, such transformations vary widely: when fermenting perilla leaves with *Lactobacillus* spp., total flavonoids increased and then declined (Wang Z. et al., 2022), and tobacco leaves with *Bacillus amyloliquefaciens* ZH-2 resulted in a significant reduction of alkaloid content (Zhou H. et al., 2025; Zhou Y. et al., 2025). All these results indicate that the interaction of specific functional microorganisms and the substrate is the key factor for fermentation success and product quality.

Hua Feng Dan (HFD) is a traditional Chinese medicine formula of more than 300 years derived from the secret recipe of the Liao family. It is used in clinical practice for wind-phlegm obstruction, stroke-induced hemiplegia and epilepsy (Xiang et al., 2016; Xu et al., 2017). Flavonoids are known as important pharmacodynamic components in HFD (Yuan et al., 2025). Its sovereign medicinal material, Hua Feng Dan Yao Mu (HFDYM), is composed of *Aconiti radix* (Chuanwu), *Pinelliae rhizome* (Banxia), *Arisaematis rhizome* (Tiannanxing), *Typhonii rhizome* (Baifuzi), and *Curcumae radix* (Yujin), combined with cattle bile and medicated leaven (Shenqu), and processed through a unique fermentation procedure (Liu, 2015; Zhang et al., 2016). This fermentation process is critical to achieve its clinical characteristic “detoxification while improving efficacy.” Although some research has focused on HFDYM, the relationship between the microbial community succession and active ingredients remains unclear. This lack of clarity hinders the controllability of fermentation. Moreover, the endpoint of fermentation is still determined by experimental and sensory methods. This creates a problem for production standardization and consistent product quality. Addressing these scientific questions is essential for modern quality control and sustainable industrial development of HFD.

This study systematically investigated the dynamic changes during the fermentation of HFDYM by analyzing three critical time points (0, 45, and 90 days). Through integrated multi-omics approaches, we characterized the temporal profiles of metabolic products and microbial communities. Our work specifically elucidated the synergistic interactions between dominant microbial populations and critical differential flavonoid metabolites. Furthermore, we isolated two main types of fermentation microorganisms (namely *Bacillus amyloliquefaciens* and *Aspergillus niger*), which both originated from HFDYM. We conducted single-strain fermentation on them to investigate their impact on the changes in flavonoid components in HFDYM. Although there have been studies exploring the correlation between microbial changes and chemical composition variations during the fermentation process of TCM, there is still a lack of research on how specific microorganisms transform a certain type of medicinal component and on systematically evaluating their impact on the composition through a single-strain fermentation system. This study will provide new scientific basis for the analysis of the fermentation mechanism of TCM and the optimization of the process.

## 2 Methods

### 2.1 Fermentation of HFDYM and sample collection

The fermentation method for HFDYM referred to the laboratory’s previously screened procedure. The 27 kg unfermented HFDYM mixture [*Typhonii rhizome* (Baifuzi)∶*Pinelliae rhizome* (Banxia)∶*Arisaematis rhizome* (Tiannanxing)∶*Aconiti radix* (Chuanwu)∶*Curcumae radix* (Yujin)∶*Medicated leaven* (Shenqu)∶ cattle bile = 2∶2∶2∶2∶1∶0.1∶8.98], provided by Zunyi Liao Yuan He Tang Pharmaceutical Co., Ltd., was divided into 9 portions, each weighing 3 kg. The experiment was divided into three groups (A0, B45, and B90), with each group performing three parallel replicates. A0 denotes unfermented HFDYM, B45 refers to fermentation for 45 days, and B90 indicates fermentation for 90 days. Upon completion of fermentation for each group, samples were transferred into sterile centrifuge tubes under aseptic conditions and subsequently stored at −80 °C until further analysis.

### 2.2 Measurement of pH and RGB color values

The pH value of HFDYM was determined in accordance with the pH determination method outlined in Part Four of the Chinese Pharmacopoeia, General Rule 0631, 2025 edition. For pH measurement, 1.0 g of the sample was suspended in 10 mL of ultrapure water, and the pH was determined with a pH meter (Mettler Toledo, FE28, Switzerland). Capture images of HFDYM using a Canon EOS 600D camera (Jayapal et al., 2022), and then analyze the RGB values of the images using ImageJ (Version 1.53t) (Jacinto et al., 2023).

### 2.3 Untargeted metabolomics analysis

For metabolite extraction, a 100 mg sample aliquot and a 6 mm diameter grinding bead were combined in a 2 mL centrifuge tube. To this, we added 400 μL of an extraction solution (methanol:water = 4:1, v/v) spiked with 0.02 mg/mL of the internal standard (L-2-chlorophenylalanine). The mixture was processed in a frozen tissue grinder at −10 °C and 50 Hz for 6 min. Subsequent extraction involved low-temperature ultrasonication at 5 °C and 40 kHz for 30 min. The sample was then held at −20 °C for another 30 min, followed by centrifugation at 13,000 g for 15 min at 4 °C. Finally, the resulting supernatant was carefully transferred into an injection vial for UHPLC–MS/MS analysis.

Chromatographic separation was performed on a Thermo Fisher UHPLC-Q Exactive HF-X system equipped with an ACQUITY BEH C18 column (100 mm × 2.1 mm i.d., 1.7 μm; Waters, United States). The mobile phase consisted of (A) water with 2% acetonitrile and 0.1% formic acid, and (B) acetonitrile with 0.1% formic acid. The analysis used a 3 μL injection volume, with the column temperature maintained at 40 °C and a constant flow rate of 0.4 mL/min. The elution gradient was programmed as follows: 0–0.5 min, 98–98% A; 0.5–7.5 min, 98–65% A; 7.5–13 min, 65–5% A; 13–14.4 min, 5–5% A; 14.4–14.5 min, 5–98% A; 14.5–16 min, 98–98% A.

Mass detection was carried out in the range of 70–1,050 m/z. The ion source conditions were as follows: sheath gas flow, 50 arb; auxiliary gas flow, 13 arb; heater temperature, 450 °C; and capillary temperature, 320 °C. Electrospray ionization (ESI) was employed in both positive and negative modes, with spray voltages set at 3500 V and −3,000 V, respectively. Full scans were acquired at a resolution of 70,000.

### 2.4 Untargeted metabolomics data analysis

The raw data matrix was generated by processing raw data through Progenesis QI metabolomics software (Waters Corporation, Milford, United States), which performed baseline filtering, peak identification, integration, retention time correction, and peak alignment. All experimental samples were run in parallel and in triplicate. Subsequent metabolomics analysis utilized the ropls R package (V1.6.2) for PCA, differential metabolite examination, and VIP analysis.

### 2.5 Microbial high-throughput sequencing analysis

The microbial genomic DNA of HFDYM was extracted using the FastPure Soil DNA Isolation Kit (MJYH, Shanghai, China). The quality and concentration of the DNA were assessed through 1.0% agarose gel electrophoresis and a NanoDrop^®^ ND-2000 spectrophotometer (Thermo Scientific Inc., United States). The V3–V4 region of the 16S rRNA gene was amplified with primers 338F (5’-ACTCCTACGGGAGGCAGCAG-3′) and 806R (5’-GGACTACHVGGGTWTCTAAT-3′), while the ITS2 region of fungi was amplified with primers ITS3F (5’-GCATCGATGAAGAACGCAGC-3′) and ITS4R (5’-TCCTCCGCTTATTGATATGC-3′). Purification and quantification of PCR products were performed using a kit and Synergy HTX instrument (Biotek, United States), respectively. High-throughput sequencing analysis of purified PCR products was conducted on an Illumina NextSeq 2000 PE300 platform (Illumina, San Diego, United States).

### 2.6 Microbiomics data analysis

Raw sequences were processed using FLASH (v0.19.6) and QIIME2 (v2020.2), with bacterial ASV sequences annotated using the SILVA 16S rRNA database (v138) and fungal ASV sequences compared against the UNITE database (v9.0). Mothur (v1.30.2) was utilized for a diversity analysis, while the ropls R package (v3.3.1) was employed for b diversity assessment, community composition evaluation, and LEfSe analysis.

### 2.7 Isolation and identification of microorganisms in HFDYM

HFDYM sample (0.5 g) of fermented for 90 days was vigorously shaken in 4.5 mL of sterile water for 30 min. The bacterial suspension was then diluted to 1,000-fold using the 10-fold serial dilution method. The diluted suspension (100 μL) was evenly spread onto Potato Dextrose Agar (PDA) and Luria-Bertani (LB) agar plates. After continuous cultivation for 3 days at temperatures of 28 °C and 37 °C, respectively, individual colonies were selected for further purification and cultivation. DNA was isolated from individual bacterial and fungal colonies using the SPARKeasy Bacterial Genomic DNA Rapid Extraction Kit (AA0202-B) and the SPARKeasy Fungal Genomic DNA Rapid Extraction Kit (AA0402-B), respectively. The collected DNA served as the template for amplification of bacterial and fungal genes using primers 27F/1492R and ITS1/ITS4, respectively, on a PCR instrument (ProFlex, Life technologies, United States). The amplified PCR products were subjected to Sanger sequencing to obtain sequences. The sequences were compared against the NCBI (National Center for Biotechnology Information) database to identify microbial species and construct a phylogenetic tree.

### 2.8 Single-strain fermentation of HFDYM

A single strain was added to sterilized HFDYM and unsterilized HFDYM for fermentation. A portion of the HFDYM sample fermented for 0 days was subjected to radiation sterilization at a dose of 16 kGy for 60 h. The radiation-sterilized HFDYM (0 days fermentation) and the non-sterilized HFDYM (0 days fermentation) were divided into 6 groups: Group W (non-sterilized HFDYM), Group M (radiation-sterilized HFDYM), Group W + A-3 (non-sterilized HFDYM supplemented with *A. niger*), Group M + A-3 (radiation-sterilized HFDYM supplemented with *A. niger*), Group W + B-7 (non-sterilized HFDYM supplemented with *Bacillus amyloliquefaciens*), and Group M + B-7 (radiation-sterilized HFDYM supplemented with *B. amyloliquefaciens*), with three parallels per group. The inoculation volume of microorganisms in each group was 3%. After fermentation at 35 °C for 7 days, changes in pH, total flavonoids and 10 flavonoids were detected.

### 2.9 Quantification of total and 10 individual flavonoids in monoculture-fermented HFDYM

The contents of total flavonoids were analyzed following the methodologies established by Zheng et al. (2013). Two grams of the sample was ultrasonically extracted for 40 min at 55 °C using 70% ethanol as the extraction solvent. Using 5% NaNO_2_, 10% Al (NO_3_)_3_, and 4% NaOH as chromogenic agents and rutin (Chengdu Pusi Biotechnology Co., Ltd., PU0001-0025, China) as the reference standard, the absorbance of total flavonoids was measured at 510 nm.

The contents of 10 flavonoids, namely (+)-catechin, vitexin, naringin, myricetin, eriodictyol, luteolin, quercetin, naringenin, apigenin and kaempferol, in each sample were determined by high performance liquid chromatography (HPLC). The HPLC analysis was performed on a Waters e2695 separation system (Waters, United States). Chromatographic separation was achieved using a ShimNex CS C18 column (4.6 mm × 250 mm, 5 μm; Shimadzu, Japan) maintained at 37 °C. The detection wavelength was set at 215 nm. The injection volume was 10 μL. The mobile phase consisted of (A) acetonitrile and (C) 0.2% phosphoric acid in water, delivered at a constant flow rate of 1.0 mL/min. A detailed gradient elution program was employed as follows: 0–10 min, 2–5% A; 10–20 min, 5–7% A; 20–25 min, 7–10% A; 25–30 min, 10–11% A; 30–35 min, 11–11% A; 35–40 min, 11–13% A; 40–45 min, 13–13% A; 45–60 min, 13–15% A; 60–65 min, 15–18% A; 65–80 min, 18–18% A; 80–120 min, 18–20% A; 120–140 min, 20–20% A; 140–150 min, 20–22% A; 150–165 min, 22–22% A. The sample extraction procedure is detailed in Supplementary Method 1.

### 2.10 Statistical analysis

The replication numbers for the collected HFDYM samples were as follows: physicochemical properties determination (*n* = 3), metabolomics analysis (*n* = 3), microbiome analysis (*n* = 3), microbial isolation (*n* = 3), and microbial fermentation verification (*n* = 3). Analysis of the physicochemical properties data involved jamovi v2.7.15 (The jamovi project, Auckland, New Zealand) for statistical processing and GraphPad Prism v10.1.2 (GraphPad Software Inc., San Diego, CA, United States) for figure generation. Continuous variables are presented as the mean ± SEM. All datasets were first subjected to the Shapiro–Wilk test to check normality. Based on the outcome, intergroup differences for normally distributed data were evaluated with an unpaired Student’s *t*-test (two groups) or one-way ANOVA with Tukey’s *post hoc* test (≥3 groups). In all inferential analyses, statistical significance was defined as *p* < 0.05.

## 3 Results

### 3.1 Characteristics and physicochemical parameters of HFDYM fermentation

**Figure 1:**
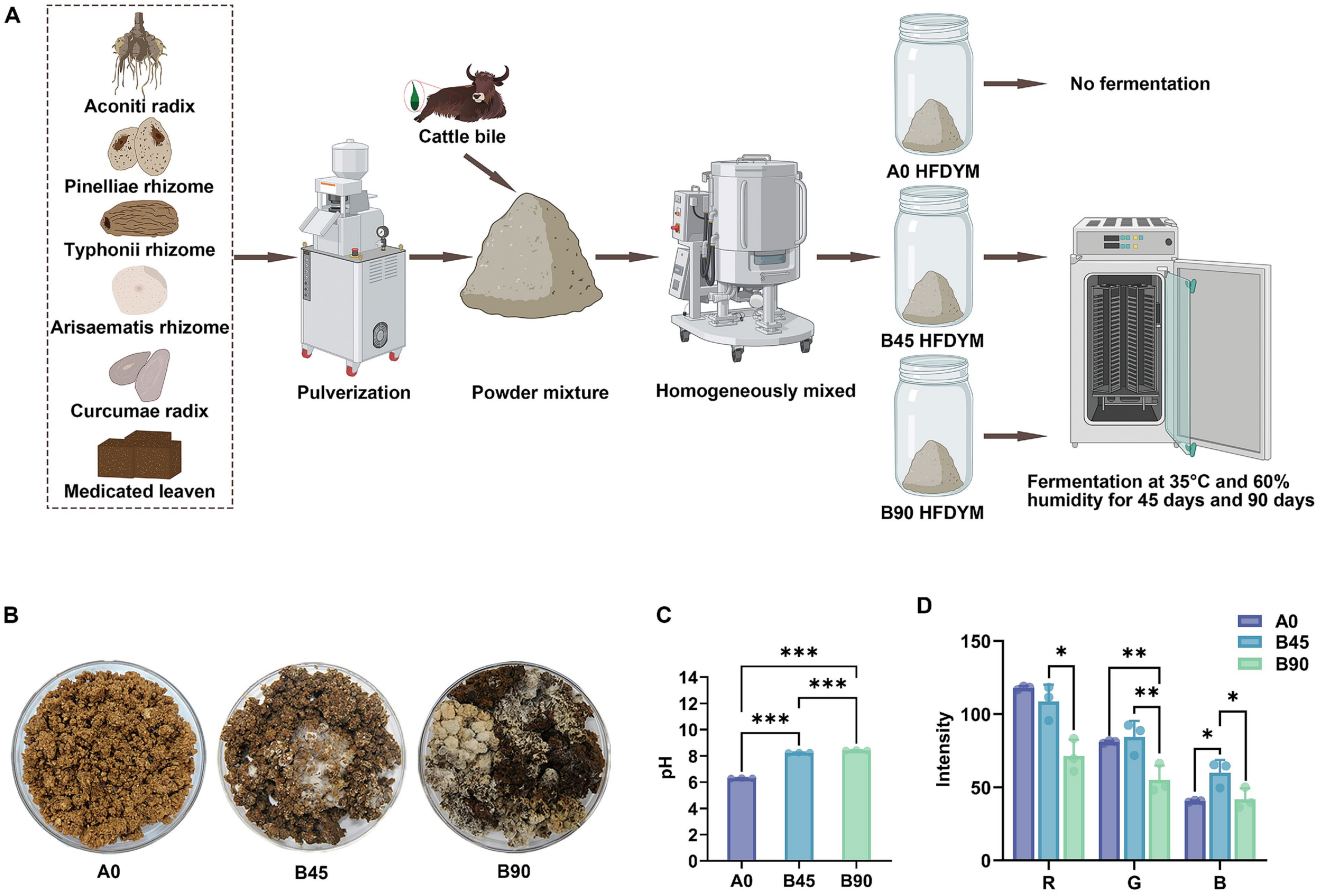
Schematic diagram of the HFDYM fermentation process and changes in its morphological characteristics and physicochemical parameters. **(A)** Schematic diagram of HFDYM fermentation, where A0 represents unfermented HFDYM, B45 represents HFDYM fermented for 45 days, and B90 represents HFDYM fermented for 90 days. **(B)** Morphological characteristics of HFDYM during three fermentation periods. **(C)** pH value of HFDYM during three fermentation periods. **(D)** RGB intensity of HFDYM during three fermentation periods. Data are mean ± SEM. *p < 0.05, **p < 0.01, ***p < 0.001.

The fermentation diagram of HFDYM is shown in Figure 1A. Morphological changes of HFDYM are shown in Figure 1A. Group A0 appeared as brown granules with slight moisture. Group B45 turned dark brown with partial agglomeration and visible white hyphae. Group B90 exhibited deepened black-brown coloration, yellowish-white colonies, and scattered reddish-brown mucus within the aggregates. Changes in microbial diversity and abundance were observed during HFDYM fermentation. Physicochemical parameters served as indicators for monitoring the fermentation process. The pH of the system rose consistently, shifting from a weakly acidic to a weakly alkaline state (*p* < 0.001) (Figure 1C). Colorimetric analysis revealed that the R intensity value of HFDYM declined significantly over time (*p* < 0.05). In contrast, the trajectories for the G (*p* < 0.01) and B (*p* < 0.05) intensity values were more complex, with both rising to a peak before subsequently declining as fermentation proceeded (Figure 1D).

### 3.2 Comparative metabolite profiling during HFDYM fermentation and screening of differential metabolites

**Figure 2:**
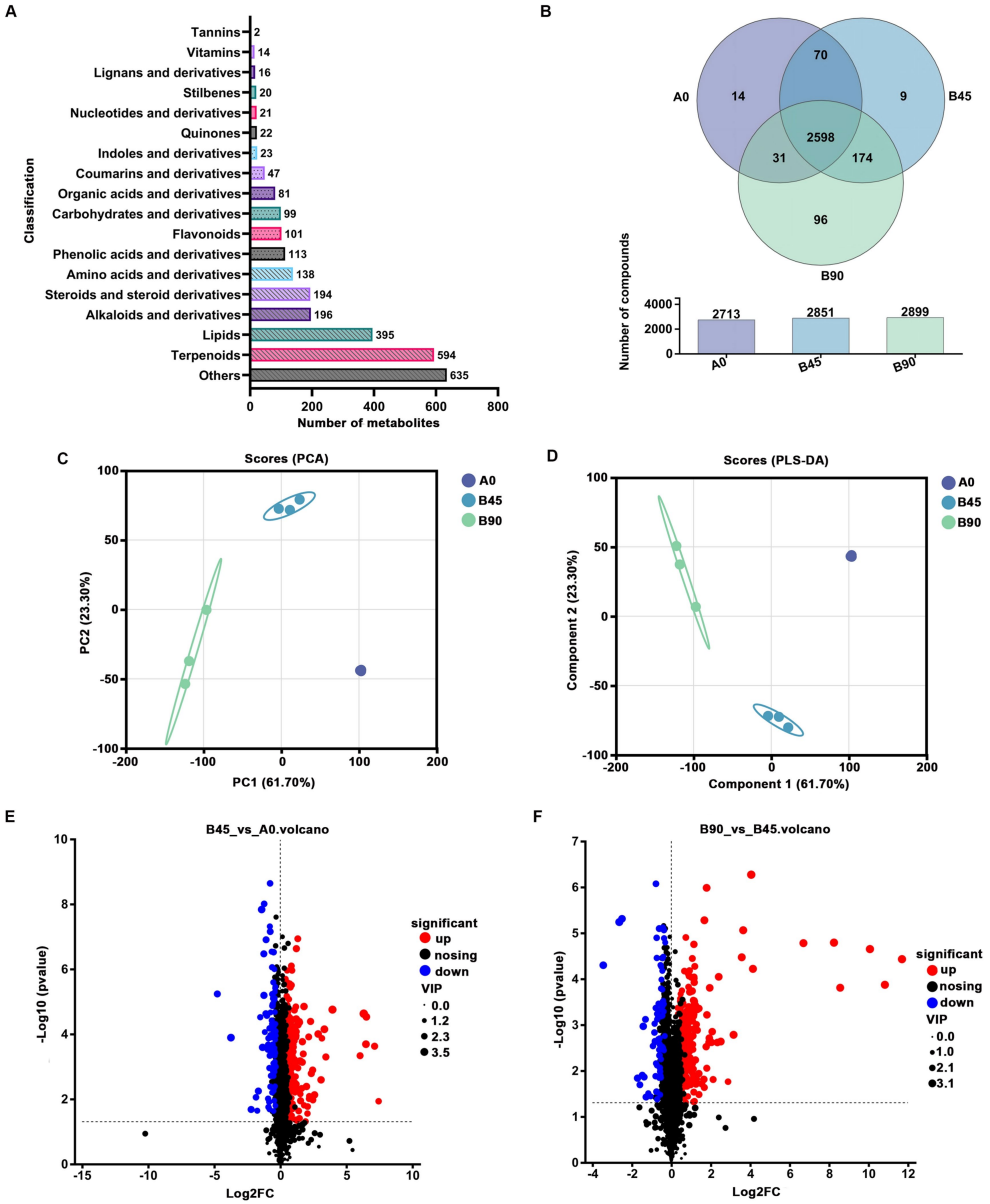
Comparison of metabolites among three fermentation periods of HFDYM based on metabolomics results. **(A)** Bar plot of total metabolite phytochemical classification; the abscissa values indicate the number of metabolites in each category, and the ordinate shows the botanical classification names of the metabolites. **(B)** Components of HFDYM during three fermentation stages Venn diagram. **(C)** PCA score plot. **(D)** PLS-DA score plot. **(E)** Volcano plots of differential metabolites for the B45_vs_A0. **(F)** Volcano plots of differential metabolites for the B90_vs_B45.

We performed untargeted metabolomic analysis using UHPLC–MS/MS to identify the chemical components during the fermentation process. Total ion chromatograms of quality control (QC) samples in positive and negative ion modes are shown in Supplementary Figure 1. Untargeted metabolomics identified 2,992 metabolites, comprising 1,737 in positive ion mode and 1,255 in negative ion mode. These included 594 terpenoids, 196 alkaloids and derivatives, 194 steroids and steroid derivatives, and 101 flavonoids, along with other compound categories (Figure 2A). A Venn diagram was used to describe the common and unique components of HFDYM at different fermentation periods. Among them, 2,598 components were common, 14 were unique to A0, 9 were unique to B45, and 96 were unique to B90 (Figure 2B). Subsequent PCA and PLS-DA analyses of sample groups (Figures 2C,D) revealed clear separation of HFDYM samples from different fermentation periods, indicating significant metabolic differences between fermentation stages. Conversely, samples from the same fermentation period clustered closely, demonstrating high metabolic consistency among intra-group replicates. Pairwise comparisons of metabolites across HFDYM fermentation groups were conducted with PCA and OPLS-DA analyses (Supplementary Figure 2). Applying screening criteria of P_value < 0.05, VIP_pred_OPLS-DA > 1, and FC > 1, 675 differential metabolites were identified, including 60 flavonoids (Supplementary Table 1). Comparisons based on OPLS-DA two-group comparative analysis; PCA and OPLS-DA score plots for the pairwise comparative analyses are shown in Supplementary Figure 2. Volcano plots were generated to visualize these results (Figures 2E,F).

### 3.3 Changes in flavonoid components, metabolic pathways and content during the HFDYM fermentation process

**Figure 3:**
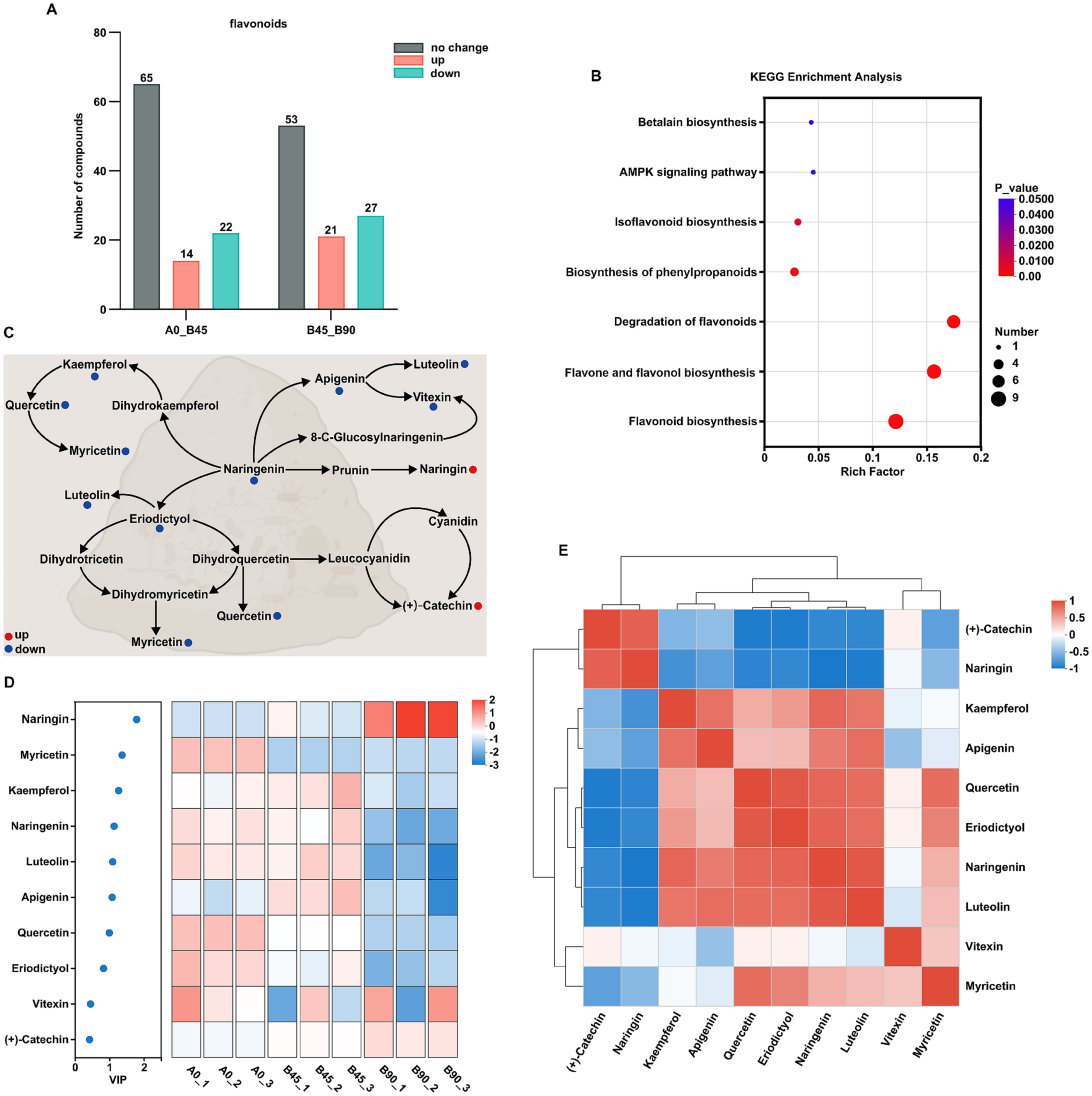
The metabolic pathway of flavonoids in HFDYM. **(A)** Bar plot showing changing trends of flavonoid differential metabolites. **(B)** KEGG enrichment scatter plot of flavonoid differential metabolites. **(C)** Conversion pathways of differential flavonoids in HFDYM. In the plot, dots represent different components: blue dots represent those that decreased in abundance during fermentation, red dots represent those that increased in abundance, and the absence of a dot indicates components that were not detected. **(D)** Analysis of VIP values for critical flavonoid constituents. **(E)** Correlation analysis of key flavonoid components.

Specifically, when comparing B45 vs. A0, 14 flavonoids were up-regulated, while 22 flavonoids were down-regulated. In the B90 vs. B45 comparison, 21 flavonoids showed up-regulation, and 27 flavonoids exhibited down-regulation (Figure 3A). KEGG enrichment analysis of differential flavonoids revealed that flavonoids were predominantly enriched in flavonoid biosynthesis, flavone and flavonol biosynthesis and degradation of flavonoids pathways (Figure 3B). Three pathways related to the synthesis of flavonoids were screened out (Supplementary Figures 3–5). The synthetic transformation pathways of the differential flavonoids in HFDYM were drawn based on the three synthetic pathways (Figure 3C), involving a total of 10 flavonoids. These 10 flavonoids were considered as the key flavonoid components in HFDYM. VIP value analysis was conducted on these 10 components, and it was found that (+)-catechin and naringin significantly increased during the fermentation process, while the rest significantly decreased (Figure 3D). Meanwhile, a correlation analysis was performed on these 10 components, and the results indicated that the correlation among them was strong, suggesting that they could mutually influence each other’s content (Figure 3E).

### 3.4 Sequencing information and diversity of microbes during HFDYM fermentation

**Figure 4:**
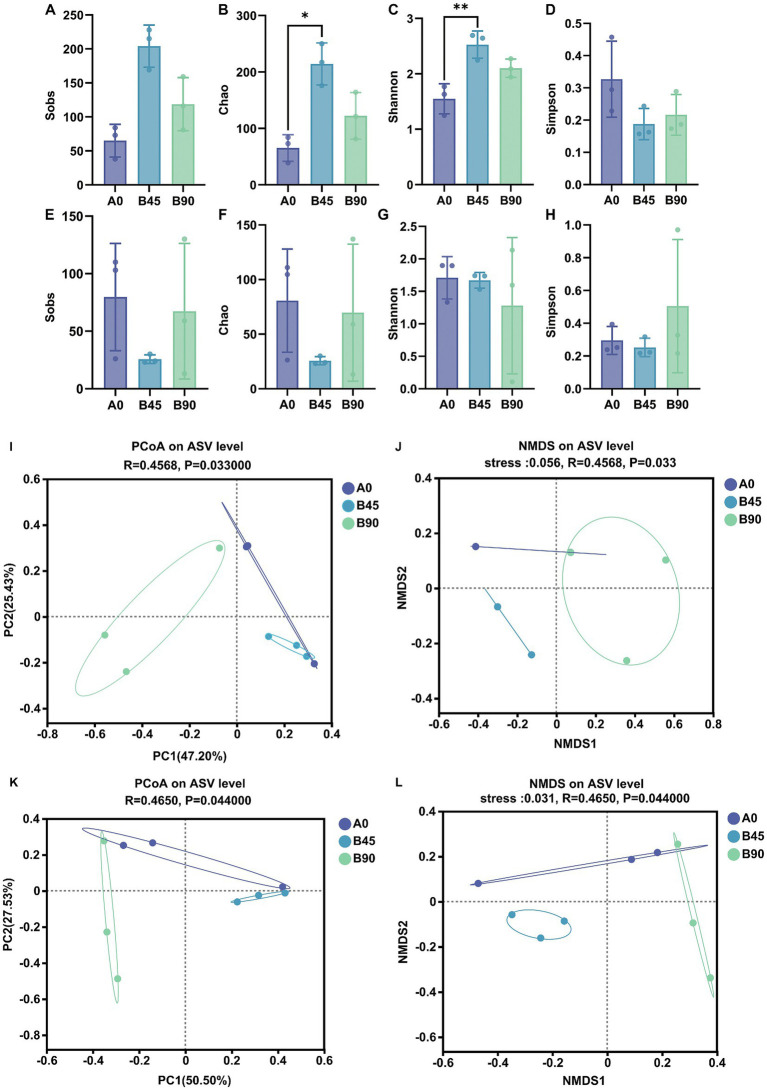
Diversity plots of HFDYM during three fermentation periods. Sobs index **(A)**, Chao index **(B)**, Shannon index **(C)**, and Simpson index **(D)** for bacteria in HFDYM at different fermentation periods. Sobs index **(E)**, Chao index **(F)**, Shannon index **(G)**, and Simpson index **(H)** for fungi in HFDYM at different fermentation periods. PCoA score plot **(I)** and NMDS score plot **(J)** (stress = 0.056) for HFDYM bacteria based on the Bray_curtis algorithm. PCoA score plot **(K)** and NMDS score plot **(L)** (stress = 0.031) for HFDYM fungi based on the Bray_curtis algorithm. Data are mean ± SEM. *p < 0.05, ** *p* < 0.01.

High-throughput sequencing of HFDYM samples at three fermentation time points yielded 315,461 (16S rRNA) and 538,268 (ITS) sequences. Analysis of sequencing depth and data volume demonstrated that the Coverage index approached 1, and rarefaction curves plateaued, confirming data reliability (Supplementary Figure 6). Sequencing results showed that the number of bacterial ASVs was the highest in the B45 group (total = 612, mean = 204), followed by B90 (total = 356, mean = 119), and the lowest in A0 (total = 195, mean = 65). Fungal ASVs were highest in the A0 group (total = 240, mean = 80), followed by B90 (total = 205, mean = 69) and lowest in the B45 group (total = 77, mean = 26) (Supplementary Table 2). Chao, Sobs, Shannon and Simpson diversity indices were calculated to measure microbial richness and diversity in different fermentation stages (Figures 4A–H). Bacterial richness (Chao and Sobs indices) initially increased and then decreased during the fermentation process. In contrast, fungal richness exhibited a consistent decline over the same period. Fungal diversity (Shannon and Simpson indices) showed fluctuations similar to its richness. As expected, both bacterial and fungal communities had dynamic changes during HFDYM fermentation. PCoA of 16S rRNA and ITS sequencing revealed that PCoA1 and PCoA2 were responsible for 72.63% variation. At the ASV level, significant differences between bacterial communities occurred in three fermentation periods (*R* = 0.4568, *p* < 0.05) (Figure 4I). For fungal communities, PCoA1 and PCoA2 account for 78.03% variation, and significant differences between periods were also observed between periods (R = 0.46650, *p* < 0.05) (Figure 4K). NMDS yielded stress values of 0.056 for bacteria and 0.031 for fungi under 0.2 indicating reliable representation of inter-sample differences (Figures 4J,L). Together, PCoA and NMDS results showed significant compositional differences between bacterial and fungal communities.

### 3.5 Microbial community composition during HFDYM fermentation

**Figure 5:**
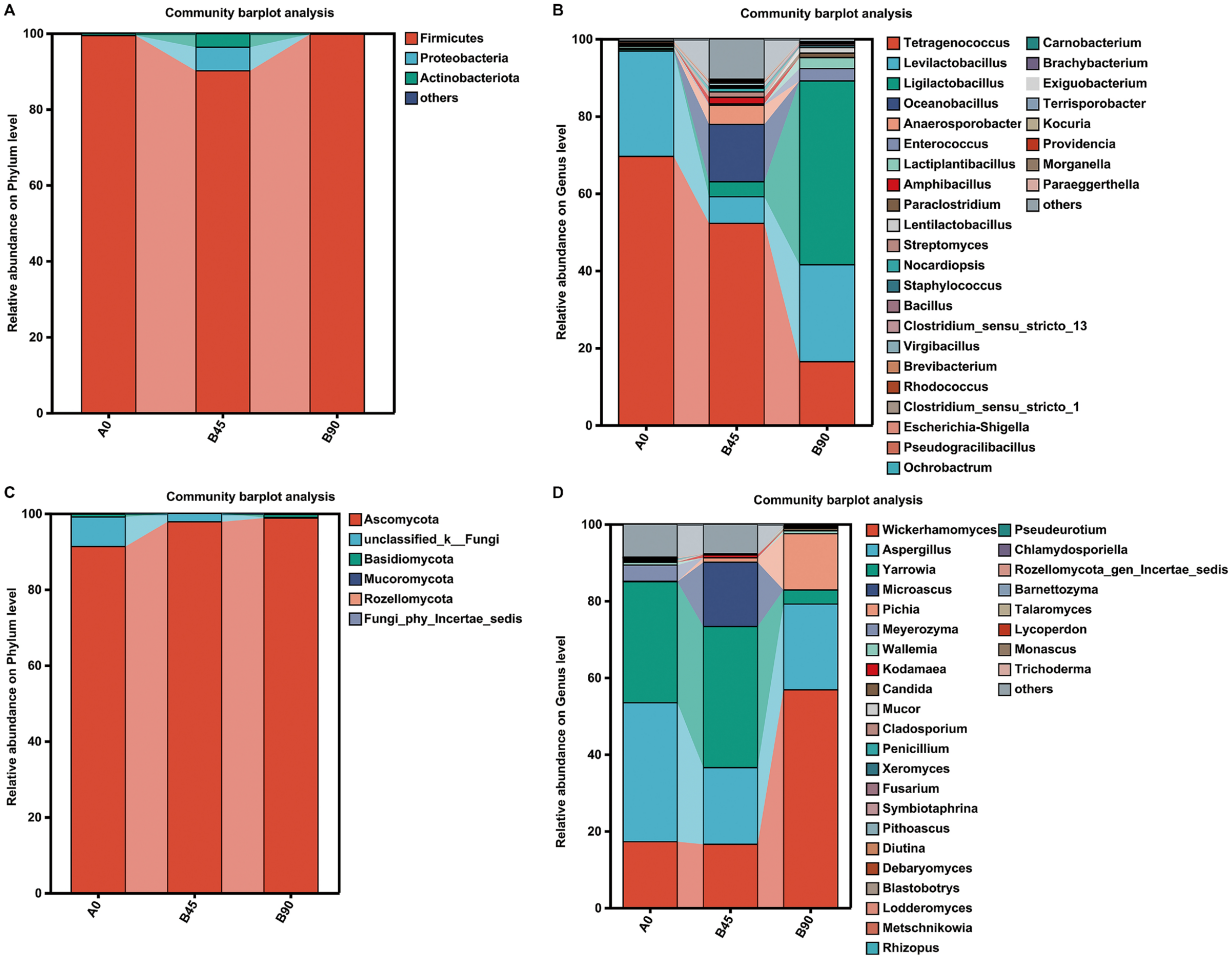
Diversity plots of HFDYM during three fermentation periods. Sobs index **(A)**, Chao index **(B)**, Shannon index **(C)**, and Simpson index **(D)** for bacteria in HFDYM at different fermentation periods. Sobs index **(E)**, Chao index **(F)**, Shannon index **(G)**, and Simpson index **(H)** for fungi in HFDYM at different fermentation periods. PCoA score plot **(I)** and NMDS score plot **(J)** (stress = 0.056) for HFDYM bacteria based on the Bray_curtis algorithm. PCoA score plot **(K)** and NMDS score plot **(L)** (stress = 0.031) for HFDYM fungi based on the Bray_curtis algorithm. Data are mean ± SEM. *p < 0.05, ** *p* < 0.01.

Firmicutes, Proteobacteria and Actinobacteria are the major groups of bacterium in HFDYM fermentation (Figure 5A). The dominant bacterium group is *Tetragenococcus*, *Levilactobacillus*, *Ligilactobacillus* and *Oceanobacillus* (Figure 5B). The fungal community is dominated by Ascomycota over all fermentation stages (Figure 5C). The major fungal genera that have high relative abundance are *Wickerhamomyces*, *Aspergillus*, *Yarrowia*, *Microascus* and *Pichia* (Figure 5D). These are the main microbiota that form the main microbiota during fermentation and likely play a role in determining final quality of HFDYM. Multi-group comparison and LEfSe found some bacterial taxa and fungal taxa (*Oceanobacillus*, *Lactiplantibacillus*, *Ligilactobacillus*, *Kodamaea* and *Pichia*) differing abundances (Supplementary Figure 7). Diversities of these species were very consistent with the microbial genus level in HFDYM.

### 3.6 Correlation analysis of dominant microorganisms with 10 differential flavonoids during HFDYM fermentation

**Figure 6:**
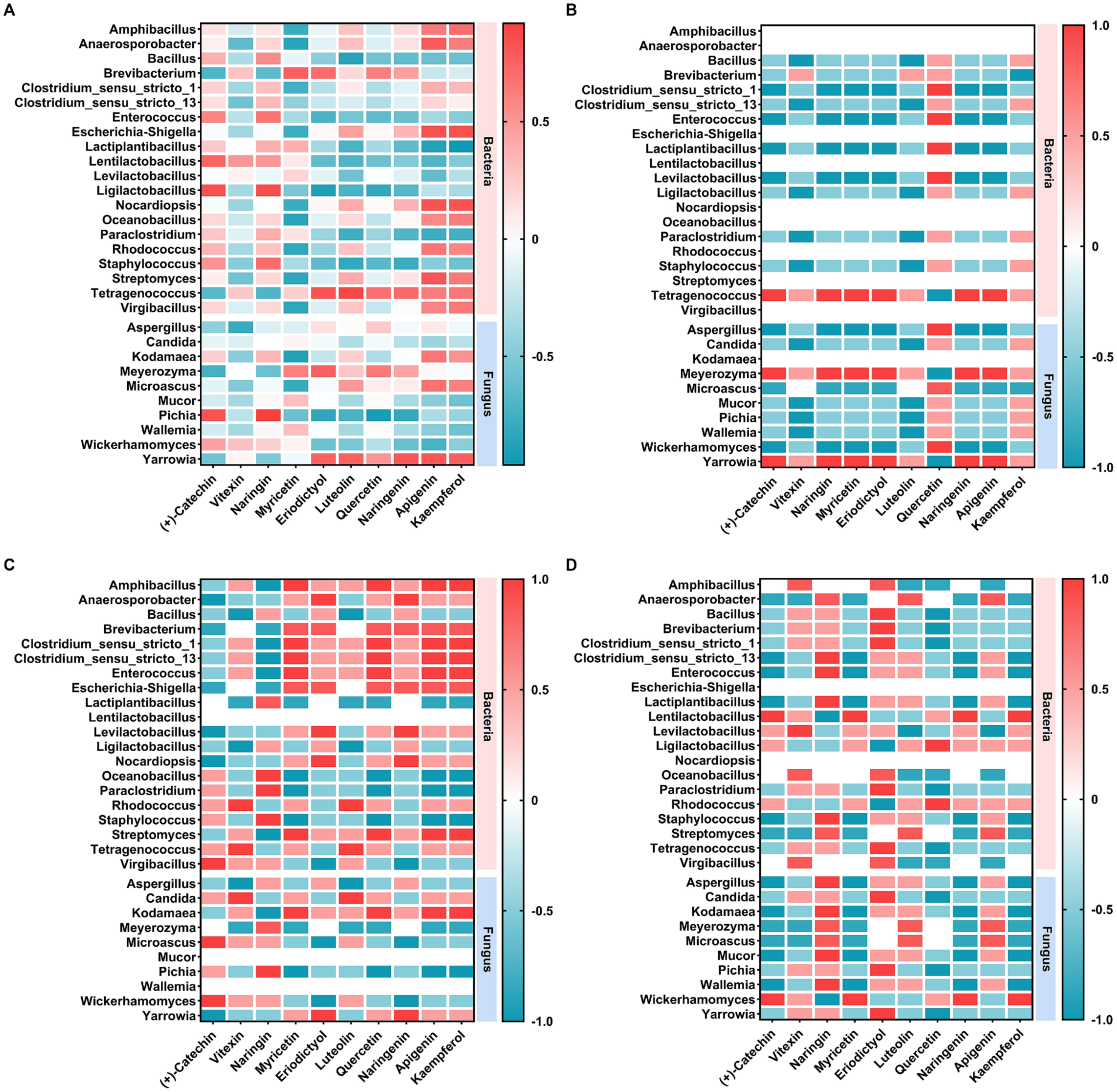
Correlation analysis heatmaps. **(A)** Heatmap of correlations between microorganisms and metabolites across three fermentation periods (20 bacterial genera, 10 fungal genera, 10 differential flavonoid metabolites). **(B–D)** Heatmaps of correlations between microorganisms and metabolites for the A0, B45, and B90 periods, respectively.

To identify microorganisms likely involved in the flavonoid accumulation in HFDYM fermentation, Spearman rank correlation analysis between dominant microorganisms and 10 different flavonoid metabolites was performed. Heatmaps on fermentation stages showed dynamic correlation patterns and stage-specific correlations (Figures 6A–D). *Aspergillus* and *Bacillus* were consistent with 10 flavonoids (mainly naringin and luteolin) during fermentation. In contrast, the correlation profile of *Aspergillus* demonstrated greater dynamism: it initially showed a strong positive correlation with quercetin, which shifted to a significant negative correlation with vitexin and luteolin during the mid-phase. By the final stage, *Aspergillus* was positively correlated with a broader spectrum of flavonoids, including luteolin and quercetin. Furthermore, *Tetragenococcus* and *Yarrowia* exhibited significant positive correlations with vitamins and eryodictyol, respectively, across all three fermentation stages. *Brevibacterium* displayed a significant negative correlation with (+)-catechin throughout the entire fermentation process. In contrast, the correlations between the remaining microbial genera and these 10 flavonoid compounds varied dynamically across the three fermentation stages. At the A0 stage, all microorganisms except *Tetragenococcus*, *Meyerozyma*, *Brevibacterium* and *Yarrowia* showed significant negative correlations with the other eight flavonoids (excluding quercetin and keampferol). However, at the B45 stage, this correlation pattern shifted markedly among these microorganisms, with many exhibiting a reversal in correlation direction, i.e., transitioning from significant negative to significant positive correlations. At the B90 stage, the correlation trends for certain microorganisms reversed again relative to those observed at the B45 stage.

### 3.7 Isolation of A-3 and B-7 and their effects on physicochemical properties of HFDYM fermentation

**Figure 7:**
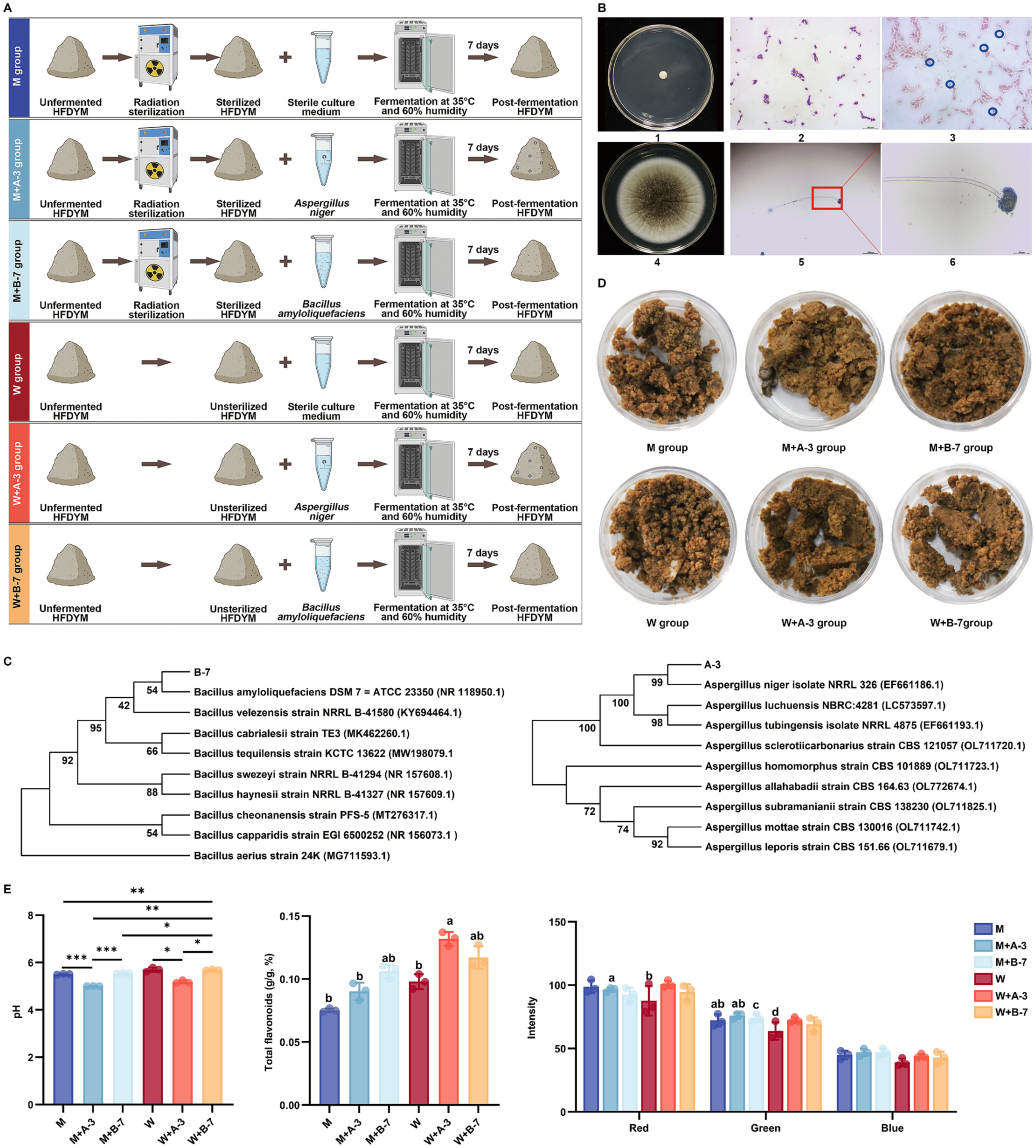
Isolation of strains A-3 and B-7 and their effects on the physicochemical properties of HFDYM fermentation. **(A)** Diagram of fermentation groups. **(B)** Growth status and micrographs of strains A-3 and B-7 identified from HFDYM after cultivation on LB and PDA media for 3 days [1–3: Morphology, Gram staining, and malachite green staining micrographs (1,000× magnification) of strain B-7, respectively; blue circles indicate malachite green-positive spore structures. 4–6: Morphology, lactophenol cotton blue staining micrograph (40× magnification), and its enlarged view (200× magnification) of strain A-3, respectively]. **(C)** Phylogenetic trees of strain B-7 and strain A-3 were constructed. DNA sequence fragments (500–600 bp) of homologous microorganisms downloaded from NCBI were used to construct the phylogenetic trees, with sequence similarity >90%. **(D)** Appearance of HFDYM inoculated with strains A-3 and B-7 after 7 days of fermentation. **(E)** Changes in physicochemical properties after fermentation with the addition of strains A-3 and B-7 were assessed. Data are mean ± SEM. *p < 0.05, **p < 0.01, ***p < 0.001. Significant differences (*p* < 0.05) are denoted by different letters **(A–D)**. All tests were one-way ANOVA with Tukey’s multiple-comparisons test.

We isolated 27 microbial strains from HFDYM samples at A0 and B90, including 16 bacteria and 11 fungi. According to microbial community composition and correlation analysis results, *Bacillus* and *Aspergillus* had high abundance and correlations with flavonoids. Therefore, isolated *B. amyloliquefaciens* and *A. niger* were used for validation experiments. Group assignment is illustrated in Figure 7A. Phylogenetic tree analysis showed that A-3 had the closest genetic relationship with *A. niger* (EF661186.1). B-7 had the closest genetic relationship with *B. amyloliquefaciens* (NR 118950.1) (Figures 7B,C). Compared with the control group without bacteria added, HFDYM inoculated with *B. amyloliquefaciens* (B-7) had no hyphae, increased pH (*p* < 0.01), lighter coloration, and a significant increase in total flavonoids (*p* < 0.01). HFDYM inoculated with *A. niger* (A-3) showed black hyphae, significantly increased total flavonoids (*p* < 0.001), significantly decreased pH (*p* < 0.001) and darker coloration (Figures 7D,E). Our findings confirmed that changes in physicochemical properties during HFDYM fermentation were caused by microbial participation.

### 3.8 Fermentation with A-3 and B-7 promoted the accumulation of bioactive flavonoids in HFDYM

**Figure 8:**
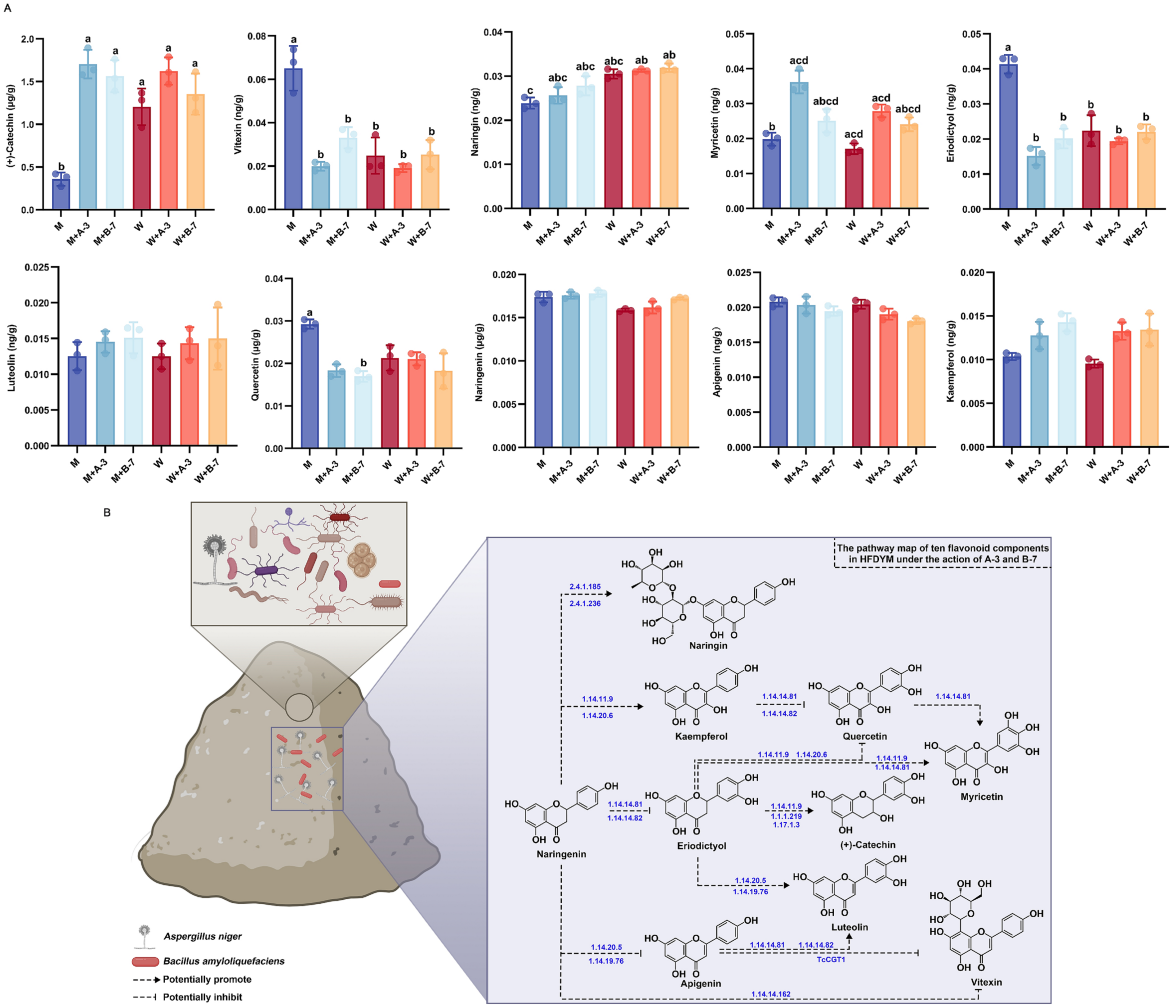
Effects of A-3 and B-7 on 10 flavonoid components in HFDYM. **(A)** Histogram comparing the content levels of 10 flavonoid components in HFDYM under the influence of A-3 and B-7. Data are mean ± SEM. Significance of data differences (*p* < 0.001) if followed by different letters above the bars. **(B)** Schematic diagram illustrating the hypothesized transformation pathways of 10 flavonoid components in HFDYM influenced by A-3 and B-7. The proposed enzymes (with EC numbers) are derived from literature annotations and the KEGG database. Direct enzymatic activities were not measured in this study. Therefore, this scheme should be considered a hypothesis-generating model rather than a validated mechanism. The EC numbers and abbreviations of the enzymes are shown in the figure, and the full names of the enzymes can be found in Supplementary Table 3.

Before single-strain fermentation, we first compared the contents of 10 flavonoids in non-sterilized and radiation-sterilized HFDYM. The results showed that radiation sterilization had no significant effect on any of the 10 components (Supplementary Figure 9). The contents of 10 flavonoid components in HFDYM fermented with strains A-3 and B-7, namely (+)-catechin, vitexin, naringin, myricetin, eriodictyol, luteolin, quercetin, naringenin, apigenin, and kaempferol, were quantitatively analyzed by HPLC. As shown in Figure 8A, after fermentation, the levels of (+)-catechin, naringin, myricetin, luteolin, naringenin, and kaempferol increased, while the contents of vitexin, eriodictyol, quercetin, and apigenin decreased. Further analysis revealed that the native microbial community in HFDYM generally exhibited synergistic effects with *B. amyloliquefaciens* and *A. niger* in the metabolism of (+)-catechin, vitexin, naringin, eriodictyol, quercetin, and apigenin. In contrast, antagonistic interactions between the native microbiota and the two inoculated strains were observed in the metabolism of myricetin, luteolin, naringenin, and kaempferol.

Based on these findings, we constructed a putative pathway for the transformation of 10 key flavonoids in HFDYM mediated by *B. amyloliquefaciens* and *A. niger* (Figure 8B). It should be noted that the enzymatic steps shown in this figure are inferred from published literature and database annotations and have not been experimentally validated in the present study. The data showed that both strains suppressed the metabolic steps converting naringenin to apigenin and eriodictyol and kaempferol to quercetin, thereby limiting the accumulation of vitexin and quercetin. They also promoted the conversion of naringenin to naringin, eriodictyol and kaempferol to myricetin, eriodictyol and apigenin to luteolin, and eriodictyol to (+)-catechin. Overall, these metabolic activities collectively facilitated the accumulation of (+)-catechin, naringin, myricetin, luteolin, and kaempferol, and accelerated the consumption of vitexin and quercetin.

## 4 Discussion

Fermentation of HFDYM serves as the core step in HFD production, during which the microbial community plays a key driving role in the biotransformation of substances. Although the effects of cattle bile, Shenqu, and specific enzymes on the composition of HFDYM have been documented in the literature (Cao et al., 2019; Cao G. et al., 2015; Cao H. et al., 2015). However, our study of dynamic monitoring of physicochemical properties, flavonoid metabolites, microbial community succession, and regulatory effects on physicochemical parameters and flavonoid component evolution during fermentation is limited. By integrating multiomics with functional validation, we have shown for the first time the collaborative interaction network between microbial community dynamics and chemical component evolution during solid state fermentation of HFDYM. We show that fermentation duration has a significant time dependent influence on the chemical composition profile and microbial community structure of HFDYM. Microbial metabolic activity appears to be a major factor influencing the chemical components.

Phenotypic traits are essential to measure the microbiology of fermentation and track fermentation progress. We documented the macroscopic nature of HFDYM throughout fermentation and quantified changes in pH and RGB values. As fermentation progressed, the color of the sample gradually moved from light brown to dark brown with more visible colonization. This color change is likely due to combination of microbial pigments created by growth and mixing of necrotic tissue with microbial pigments following herbal organic matter breakdown (Fukami et al., 2021). The pH started at 6.27, which indicates significant incorporation of weakly acidic to neutral bovine bile during HFDYM preparation (Cao et al., 2019). A sharp rise was seen during mid and late fermentation. Two possible explanations of this alkaline transition may be due to increased *Streptomyces* abundance at mid-fermentation to secrete antibiotics suppressing acid producing bacteria and reducing acid production (Jankowitsch et al., 2012); alkaline proteases released by *Bacillus* and *Aspergillus* later likely contributed to pH elevation (Hakim et al., 2018; Zhao et al., 2021). Phenotypic and physicochemical parameters were markedly altered during HFDYM fermentation. Dynamic multi-parameter monitoring results (color and pH variations) can provide a foundation for scientific determination system for HFDYM fermentation endpoint. Specific criteria for these parameters need further establish and validated by pharmacodynamic efficacy tracking studies.

Metabolomics has emerged as a powerful tool for systematically elucidating the dynamic changes of chemical constituents during the fermentation of TCM (Zhou H. et al., 2025; Zhou Y. et al., 2025). Therefore, we employed UHPLC–MS/MS-based metabolomics analysis and identified 60 differential flavonoid metabolites. In our study, the abundances of flavonoids such as mammeisin, morusinol, and naringin were significantly elevated during the fermentation of HFDYM, while the levels of other components, including heterophyllin, showed a marked decrease. Morusinol can exert antithrombotic effects by inhibiting the activation of the aIIb/b3 receptor (Kwon, 2023). Furthermore, mammeisin exhibits significant inhibitory activity against pathogenic fungi such as *Candida* sp. (Marcondes et al., 2015). Naringin exhibits neuroprotective effects in a cerebral ischemia–reperfusion injury model (Keskin et al., 2025) and cell type-specific preventive effects (Babu et al., 2025). In contrast, although heterophyllin can inhibit LPS-induced inflammation and apoptosis via the PI3K/AKT pathway (Yang et al., 2018), it has potent cytotoxicity against various tumor cells (Bortolotti et al., 2023). KEGG pathway analysis indicated that the transformation of these flavonoids mainly involves the flavonoid biosynthesis pathway, the flavone and flavonol biosynthesis pathway, and the isoflavonoid biosynthesis pathway. Specifically: naringenin can be converted into naringin under the action of UDP-glucose:flavanone-7-O-glucosyltransferase and rhamnosyltransferase (Sharma et al., 2019), and can also be transformed into eriodictyol via 4-hydroxyphenylacetate hydroxylase (Wu et al., 2022). Eriodictyol can be further transformed via two pathways: on the one hand, it is dehydrogenated at the C-ring 2,3-positions catalyzed by flavone synthase to form luteolin (Horowitz and Gentili, 1960); on the other hand, it is converted into dihydroquercetin by flavanone 3-hydroxylase (Han et al., 2017). Dihydroquercetin can be further reduced to (+)-catechin (Tanner et al., 2003; Huang et al., 2023), or converted into kaempferol and quercetin under the catalysis of flavonol synthase (Zarshenas, 2022; Tartik et al., 2023). Myricetin is synthesized through the coordinated actions of flavonoid-3′,5′-hydroxylase and flavonol synthase (Olsen et al., 2010; Marín et al., 2018). Kaempferol can be directly converted into quercetin via hydroxylation at the 3′ position of the B ring catalyzed by flavonoid 3′-hydroxylase (Jan et al., 2021). In addition, naringenin is directly dehydrogenated to generate apigenin (Wang H. et al., 2022), which is then converted into vitexin via C-glycosylation (Liu et al., 2021). Collectively, this study provides new insights into the metabolic processes and potential pharmacological functions of flavonoids in HFDYM, offering a theoretical foundation for its further development and application.

Microbial community structure analysis further revealed significant differences between HFDYM and other fermented TCM. In this study, high-throughput sequencing technology was used to detect microbial changes at various stages of HFDYM fermentation. Bacterial genera such as *Tetragenococcus* and *Levilactobacillus* became dominant genera in HFDYM fermentation, both belong to lactic acid bacteria and are commonly used in the production of sauerkraut, sour bamboo shoots, dairy products, etc. (Wu and Shah, 2017; Yao et al., 2021; Fan et al., 2023; Li et al., 2023; Verni et al., 2023; Tang et al., 2024; Wang et al., 2024; Wen et al., 2024). In other fermented TCM, the dominant bacterial genera are mainly *Bacillus* (Ding et al., 2024; Guo et al., 2016; Ma et al., 2022) but in this study, large amounts of *Levilactobacillus* and *Tetragenococcus* bacteria were detected both before and after fermentation, indicating that the fermentation of HFDYM differs significantly from other fermented TCM. This may be due to the addition of large amounts of cattle bile in HFDYM fermentation. Cattle bile contains large amounts of bile acids (Chen et al., 2022), thus making the fermentation matrix acidic, which in turn leads to a higher proportion of acid-tolerant bacteria. Fungal genera such as *Wickerhamomyces* and *Aspergillus* became dominant genera in HFDYM fermentation. The diversity of medicated leaven at different fermentation times was analyzed using PCR-DGGE technology, and it was found that *Aspergillus* is one of the fungi present in its fermentation process (Liu et al., 2017). Analysis of the microbial community of medicated leaven using high-throughput sequencing technology revealed that *Aspergillus* and *Wickerhamomyces* are the top-ranked dominant genera during fermentation, with *Aspergillus* being the dominant genus in both early and mid-late fermentation stages (Ding et al., 2024). During the processing of *Pinelliae Fermentata* (Banxiaqu), dominant fungi were isolated and identified, and it was found that *Aspergillus* also acts as a dominant genus (Guo et al., 2016).

Notably, the decrease in flavonoids such as naringenin, eriodictyol, apigenin and vitexin observed during fermentation may be related to microbial activities. In addition to *Bacillus amyloliquefaciens* possessing naringinase and glycosyltransferase activities (Zhu et al., 2017; Koirala et al., 2019) and *Aspergillus niger* catalyzing deglycosylation and C-ring cleavage (Cao G. et al., 2015; Cao H. et al., 2015; Bentouhami et al., 2026). Beyond these two strains, other core genera also contribute. Among bacteria, *Tetragenococcus halophilus* encodes a complete flavonoid degradation pathway and produces *β*-glucosidase; *Levilactobacillus brevis* and *Oceanobacillus zhaokaii* harbor similar degradation pathways encoding glycosyl hydrolases. Among fungi, *Wickerhamomyces anomalus* produces β-glucosidase and esterase, reducing apigenin content during prolonged fermentation (Palmer et al., 2020); *Yarrowia lipolytica* can convert naringenin to apigenin and degrade isoflavones; and *Pichia guilliermondii* efficiently secretes isoflavone β-glycosidase (So et al., 2010). In summary, the coordinated activities of these microbial enzymes are consistent with a possible role in the reduction of flavonoids observed during HFDYM fermentation.

To directly verify the effects of microorganisms on physicochemical properties and key flavonoid components, we performed correlation analysis and further functional validation through isolation and cultivation experiments. Correlation analysis showed certain associations between microorganisms and physicochemical parameters as well as flavonoid metabolites. Subsequently, culturable dominant strains—*B. amyloliquefaciens* and *A. niger*—were isolated from HFDYM and used in back-inoculation fermentation experiments. The results indicated that both strains significantly affected pH, RGB values, total flavonoid content, and the levels of 10 key flavonoid components. Studies have shown that *B. amyloliquefaciens* has the ability to produce various active enzymes (Yang et al., 2016; Zalila-Kolsi et al., 2023). Among them, strains W25 and P29 can significantly increase soil pH (Xiong et al., 2022), and strains SRCM 100730 and SRCM 100731 significantly increase total isoflavone content during soybean fermentation (Jeong et al., 2020), which aligns with the findings of this study. *A. niger* produces citric acid during fermentation, leading to a decrease in pH (Meijer et al., 2011; Behera, 2020), and as a fungal elicitor, it can significantly increase the total flavonoid content in adventitious roots of *Glycyrrhiza uralensis* and in summer green tea (Li et al., 2016; Cai et al., 2023). Furthermore, *Aspergillus niger* can release (+)-catechin and eriodictyol (Espitia-Hernández et al., 2022; Tian et al., 2023) and convert naringenin into kaempferol (Liao et al., 2023).

This study has several limitations. Only three time points were analyzed, which may not fully capture the dynamic changes during fermentation. The single-strain validation experiments were conducted for only 7 days, which may not fully reflect the outcomes of the real fermentation process. In future studies, we will extend the fermentation duration and optimize the fermentation process. Furthermore, this study did not directly quantify toxic aconitum alkaloids, although our previous work under the same fermentation conditions has systematically reported their significant reduction (Cao et al., 2020; Shu et al., 2026). In sum, we found that *B. amyloliquefaciens* and *A. niger* significantly influence the physicochemical properties and accumulation of key flavonoids during fermentation. The metabolic activity of these microorganisms can not only promote the accumulation of pharmacologically active flavonoids but also enhance the bioactivity of HFDYM, supporting the further development and application of flavonoids.

## 5 Conclusion

This study employed an integrated multi-omics approach to investigate the relationship between microbial community dynamics and flavonoid transformation during the fermentation of HFDYM. The results demonstrated that dominant genera such as *Bacillus* and *Aspergillus* were significantly correlated with changes in 10 key flavonoid components. Functional validation through single-strain fermentation further indicated that *B. amyloliquefaciens* and *A. niger* played a significant role in promoting the generation of flavonoids such as naringin, kaempferol, and myricetin, while accelerating the degradation of vitexin, eriodictyol, and quercetin. These findings provide preliminary correlational evidence linking changes in flavonoid components to specific microorganisms, offering a hypothesis-generating basis for monitoring and optimizing fermentation processes in traditional medicine production.

## Data Availability

The raw reads of the microbiome were deposited in the NCBI Sequence Read Archive (SRA) database (accession number: PRJNA1290699.
