# Supplementary material for: Microbe-driven flavonoid biotransformation revealed by multi-omics analysis during Hua Feng Dan Yao Mu fermentation
Source: Front Microbiol. 2026 Jun 17;17:1816282. doi: 10.3389/fmicb.2026.1816282 (PMC13351095; doi:10.3389/fmicb.2026.1816282)
Supplement: Supplementary file 1 [file Data_Sheet_1.docx]

**Supplementary material**

**Microbe-Driven Flavonoid Biotransformation Revealed by Multi-Omics Analysis During Hua Feng Dan Yao Mu Fermentation**

**Supplementary material contains:**

Supplementary Figs. 1-9

Supplementary Tables 1-3

Supplementary Methods 1


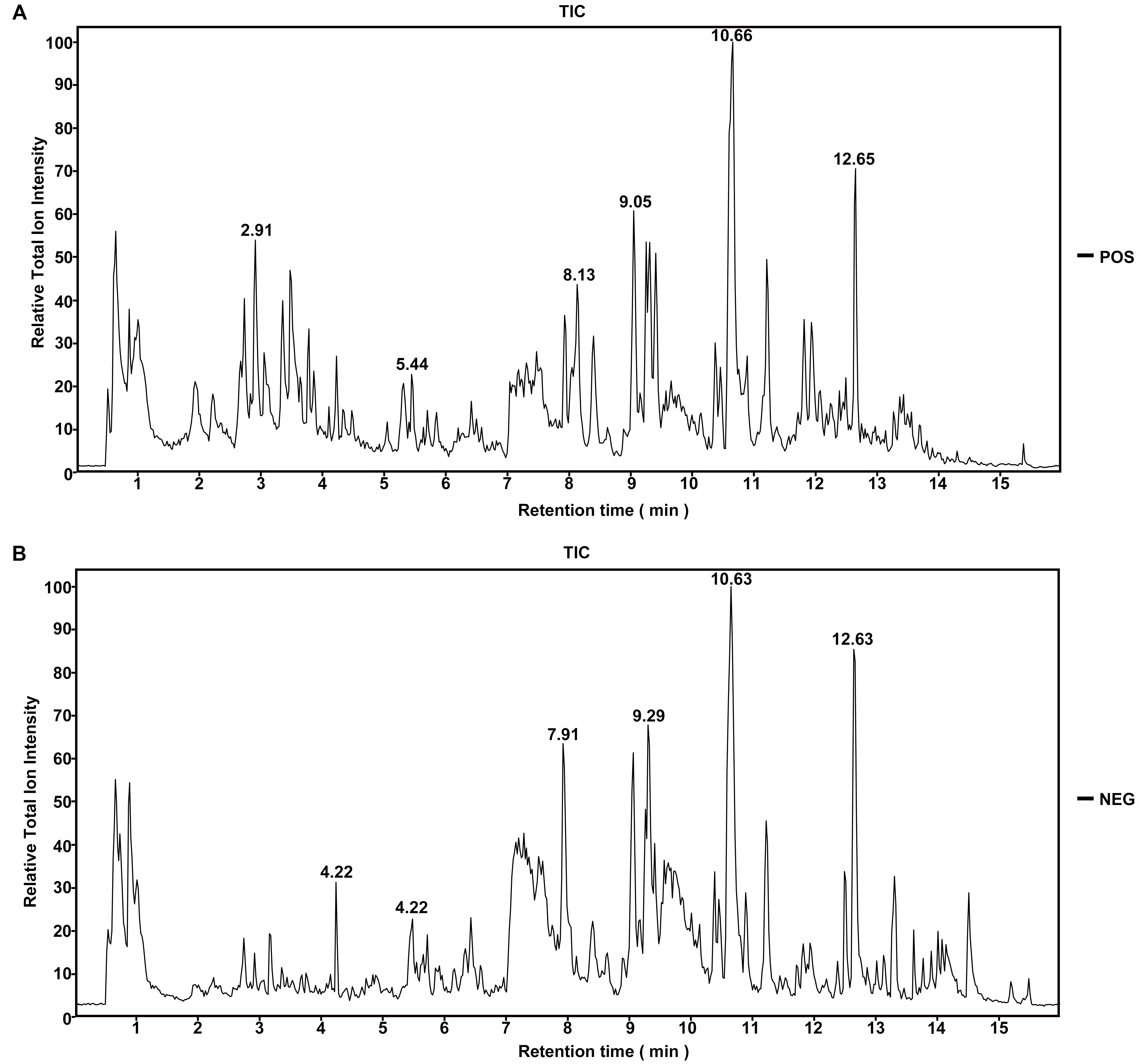


**Supplementary Fig. 1** Total ion chromatograms (TIC) of quality control samples for HFDYM across the three fermentation periods involved in this study. **(A)** Positive ion mode; **(B)** Negative ion mode.


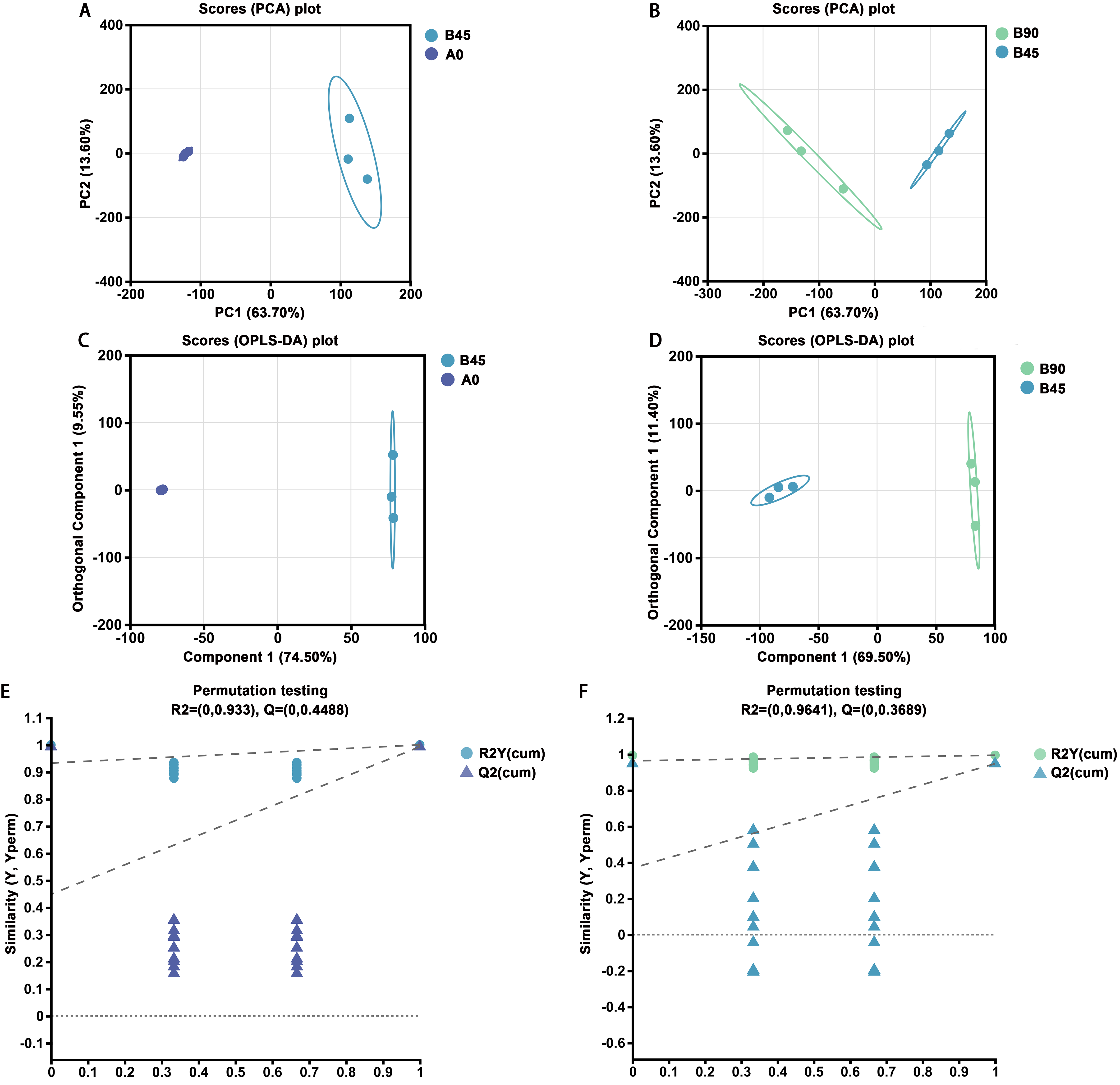


**Supplementary Fig. 2** Pairwise comparative analysis of metabolomics data for HFDYM samples across three fermentation periods. **(A)** PCA score plot for the B45_vs_A0 comparison. **(B)** PCA score plot for the B90_vs_B45 comparison. **(C)** OPLS-DA score plot for the B45_vs_A0 comparison. **(D)** OPLS-DA score plot for the B90_vs_B45 comparison. **(E)** OPLS-DA model validation plot for the B45_vs_A0 comparison, R^2^Y(cum)=1.000, Q^2^(cum)=0.993. **(F)** OPLS-DA model validation plot for the B90_vs_B45 comparison, R^2^Y(cum)=0.995, Q^2^(cum)=0.948.

**
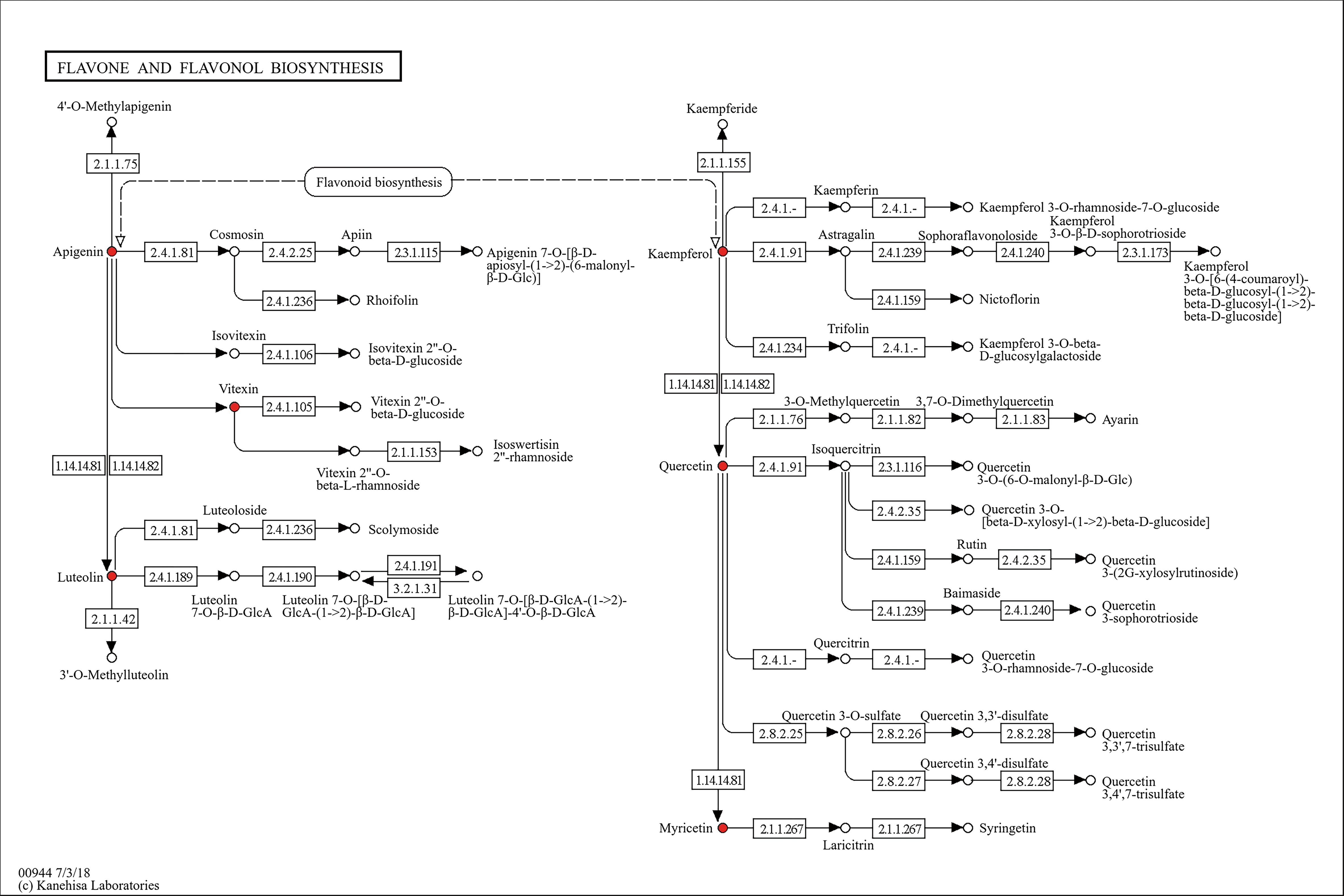
**

**Supplementary Fig. 3** Flavoneand flavonol biosynthesis pathway. The red dot denotes that the corresponding component was detected in HFDYM.

**
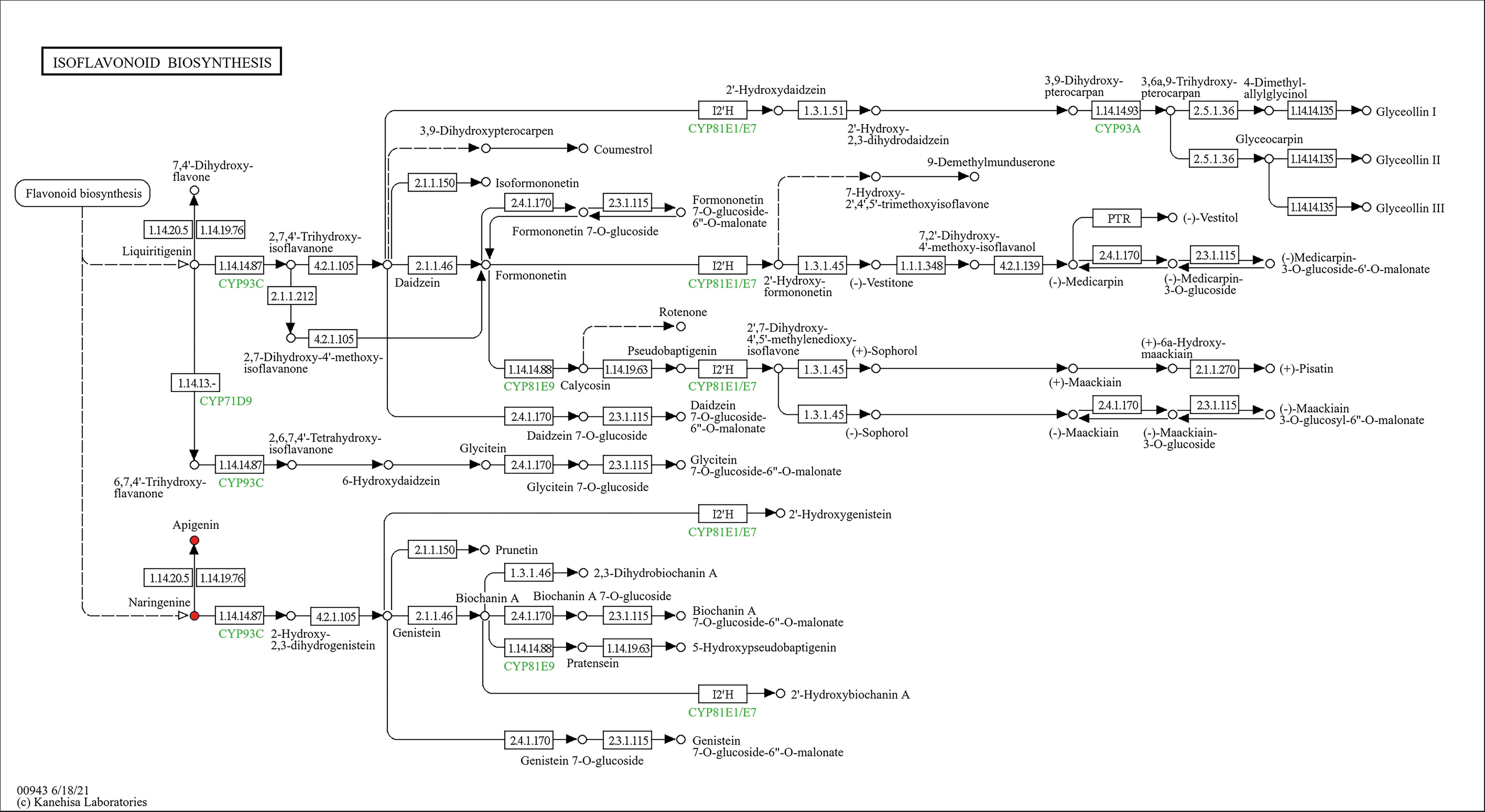
**

**Supplementary Fig. 4** Isoflavonoid biosynthesis pathway. The red dot denotes that the corresponding component was detected in HFDYM.

**
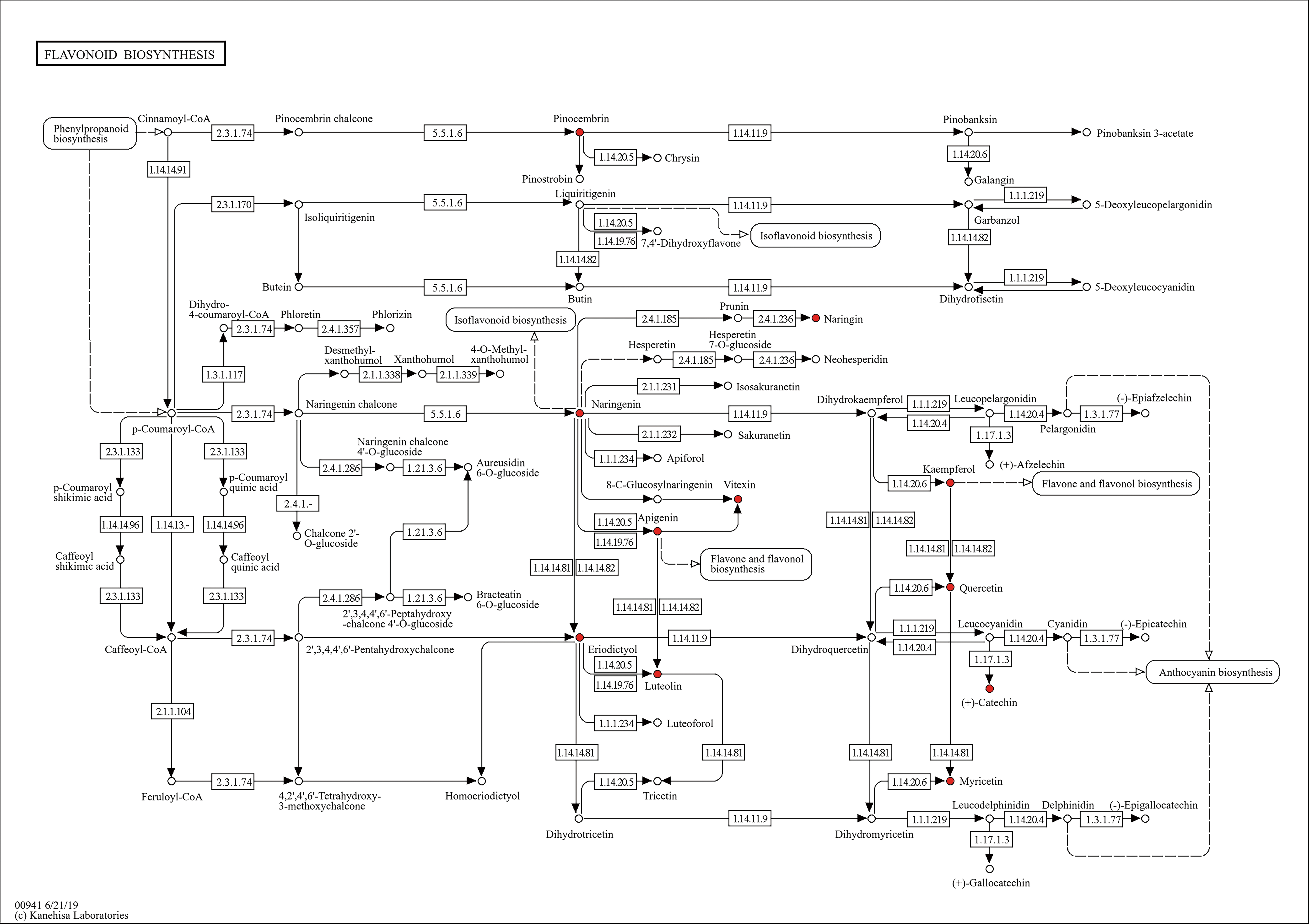
**

**Supplementary Fig. 5** Flavonoid biosynthesis pathway. The red dot denotes that the corresponding component was detected in HFDYM.


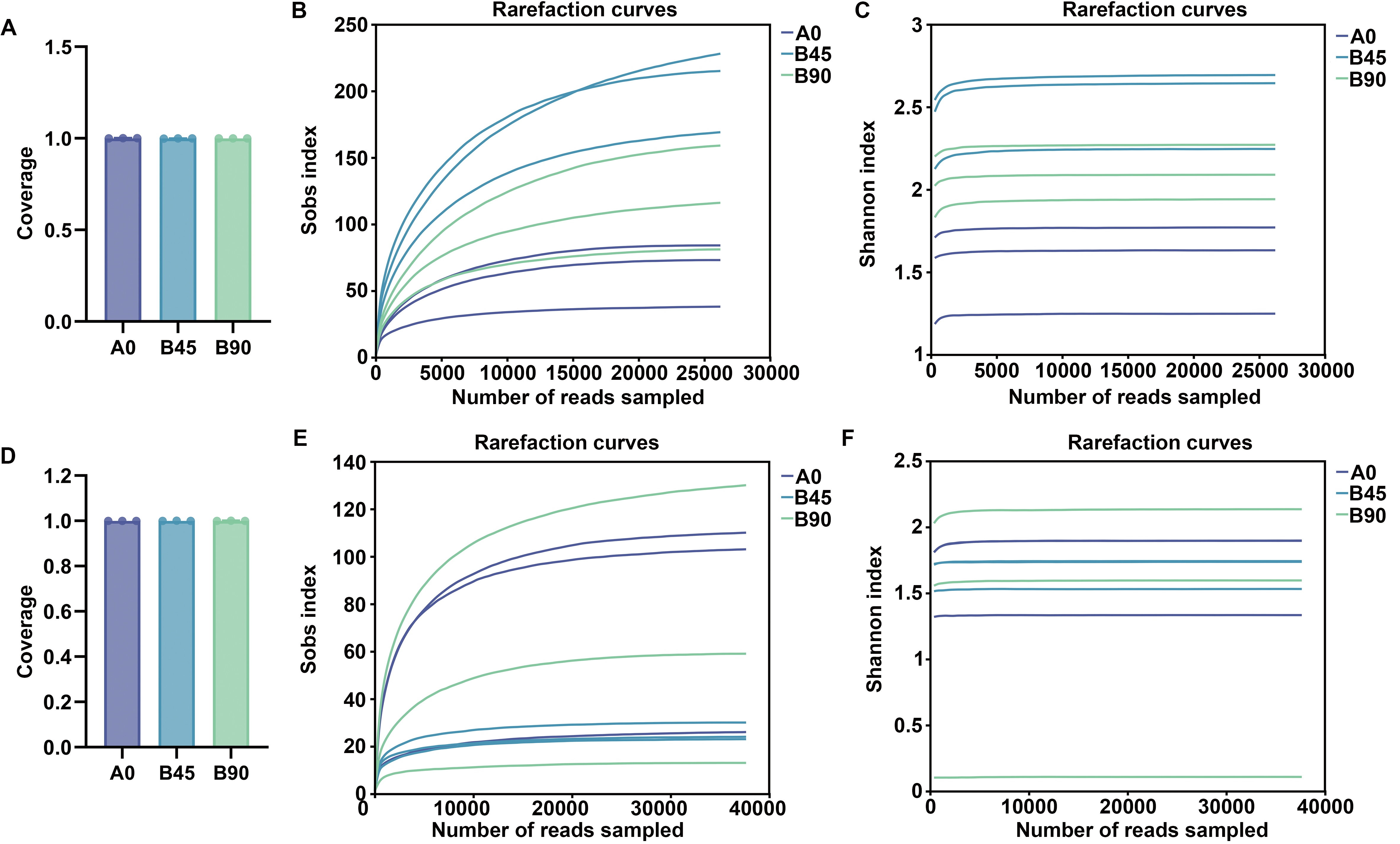


**Supplementary Fig. 6** Sequencing depth analysis of HFDYM samples from three fermentation periods. **(A)** Bar plot of bacterial Coverage index. **(B)** Bacterial rarefaction curves based on the Sobs index. **(C)** Bacterial rarefaction curves based on the Shannon index. **(D)** Bar plot of fungal Coverage index. **(E)** Fungal rarefaction curves based on the Sobs index. **(F)** Fungal rarefaction curves based on the Shannon index. Each different color represents a different fermentation period. In the bar plots, data are presented as mean ± standard error; in the rarefaction curves, each curve represents one sample. In this study, the Coverage indices for both bacteria and fungi approached 1, and the rarefaction curves plateaued, indicating that this study has captured the maximum microbial diversity of the samples, and deeper sequencing would not significantly yield new taxa.


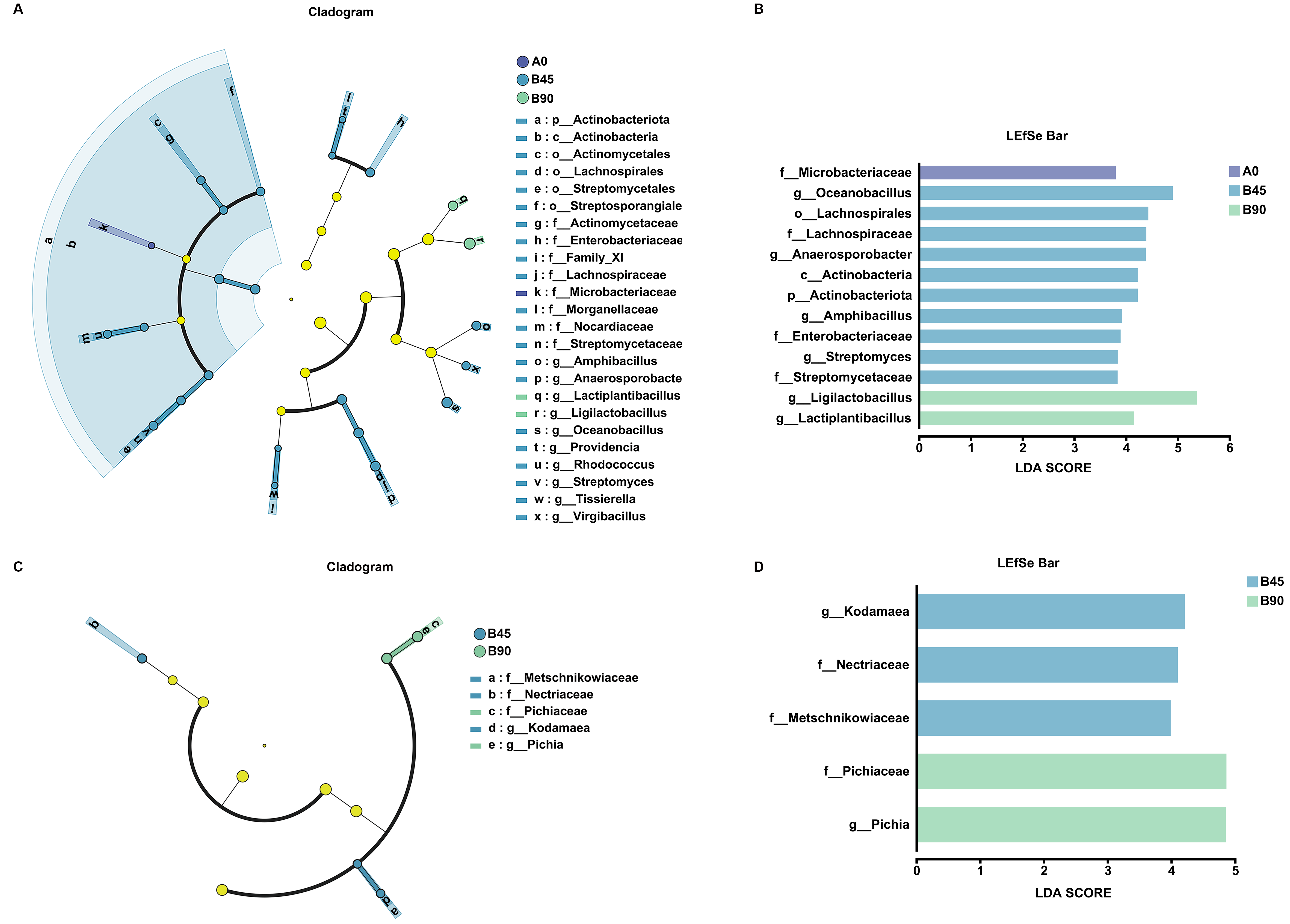


**Supplementary Fig. 7** Differential microbial community analysis of HFDYM samples across three fermentation periods using LEfSe. Microorganisms with a linear discriminant analysis (LDA) threshold greater than 3.5 were identified as key taxa for their respective fermentation time points. **(A-B)** Community differential analysis of the bacterial community. **(C-D)** Community differential analysis of the fungal community.


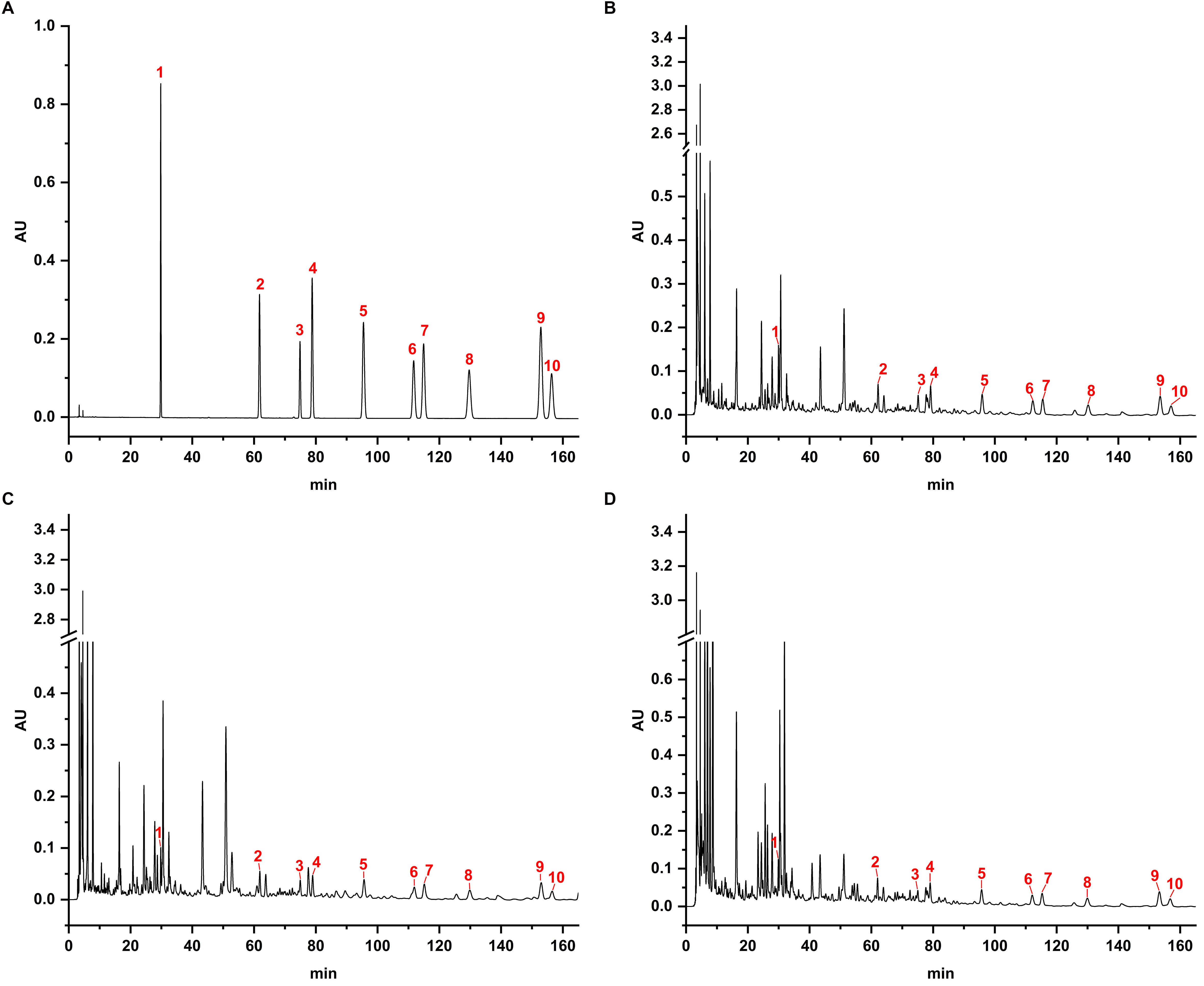


**Supplementary Fig. 8** HPLC profiling of reference compounds and test samples. **(A)** Mixed reference standards; **(B)** Test sample #1 (M+A-3 group); **(C)** Test sample #2 (W group); **(D)** Test sample #2 (M+A-3 group). Peaks are designated as follows: 1, (+)-catechin; 2, vitexin; 3, naringin; 4, myricetin; 5, eriodictyol; 6, luteolin; 7, quercetin; 8, naringenin; 9, apigenin; 10, kaempferol.


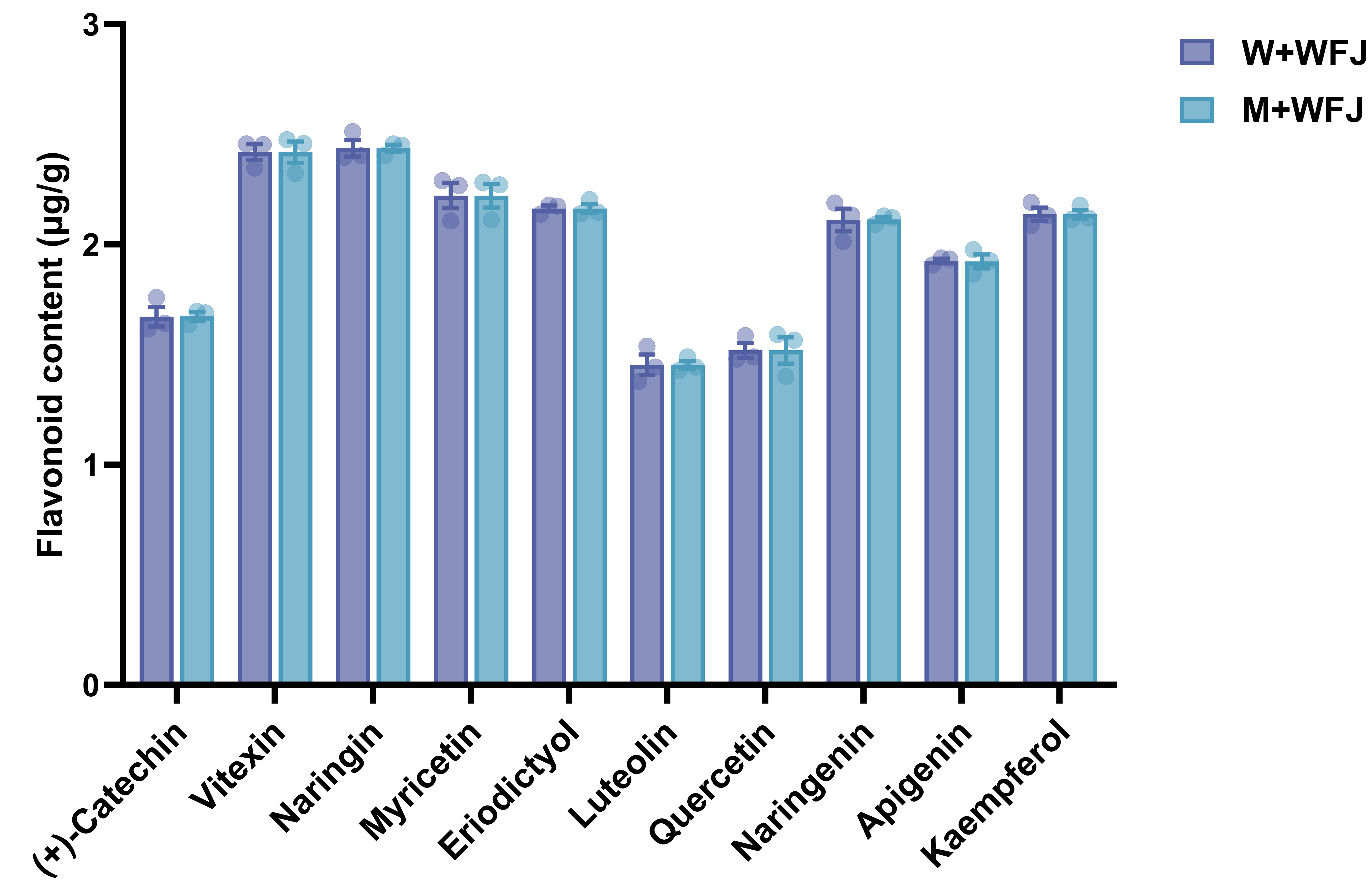


**Supplementary Fig. 9** Contents of ten flavonoids in HFDYM before and after radiation sterilization.. W+WFJ: unirradiated sterilized HFDYM; M+WFJ: sterilized HFDYM.

**Supplementary Table 1.** Differential flavonoid metabolites during the fermentation process of HFDYM.

| **No.** | **Metabolite** | **Adduct** | **Formula** | **RT (sec)** | **m/z (Da)** | **B45 / A0** | | | **B90 / B45** | | | **B45 / A0** | **B90 / B45** |
| --- | --- | --- | --- | --- | --- | --- | --- | --- | --- | --- | --- | --- | --- |
|  |  |  |  |  |  | ***p*** | **VIP** | **FC** | ***p*** | **VIP** | **FC** |  |  |
| 1 | (+/-)-2',4',5,7-Tetrahydroxy-3',8-diprenylisoflavanone | ESI+ | C25H28O6 | 5.011 | 425.1962415 | 0.2783 | 0.8604 | 1.2707 | 0.009449 | 1.8453 | 1.6253 | ↑ | ↑** |
| 2 | (7'R,8'R)-4,7'-Epoxy-3',5-dimethoxy-4',9,9'-lignanetriol 9'-glucoside | ESI- | C26H34O11 | 4.4004 | 521.2042338 | 2.706E-6 | 1.4494 | 0.7943 | 0.008937 | 0.8073 | 1.0984 | ↓*** | ↑** |
| 3 | (R)-Glabridin | ESI+ | C20H20O4 | 5.9883 | 366.169087 | 0.8079 | 0.2254 | 1.0782 | 0.03646 | 1.745 | 1.7888 | ↑ | ↑* |
| 4 | (*S*)-(*E*)-2'-(3,6-Dimethyl-2-heptenyl)-3',4',7-trihydroxyflavanone | ESI+ | C24H28O5 | 9.9901 | 793.3906766 | 0.4375 | 0.4371 | 0.9047 | 0.00124 | 2.1651 | 2.183 | ↓ | ↑** |
| 5 | (*S*)-(*E*)-8-(3,6-Dimethyl-2-heptenyl)-4',5,7-trihydroxyflavanone | ESI- | C24H28O5 | 6.2533 | 441.1923397 | 0.00126 | 1.4454 | 1.3821 | 0.02657 | 1.1916 | 1.2324 | ↑** | ↑* |
| 6 | 2''-(6-Acetylglucosyl)astragalin | ESI+ | C29H32O17 | 10.4929 | 716.1798196 | 0.004719 | 1.9228 | 1.6538 | 0.01144 | 1.5683 | 1.2965 | ↑** | ↑* |
| 7 | 3-Hydroxyglabrol | ESI- | C25H28O5 | 5.7165 | 453.1931915 | 0.009817 | 2.0819 | 4.5462 | 0.01375 | 1.9182 | 1.7087 | ↑** | ↑* |
| 8 | 3'-Methoxyapigenin | ESI- | C16H12O6 | 7.0839 | 299.0564775 | 0.04151 | 0.9912 | 1.1274 | 0.03555 | 1.1575 | 0.8319 | ↑* | ↓* |
| 9 | 6''-Acetylliquiritin | ESI- | C23H24O10 | 4.4689 | 505.1364605 | 0.001116 | 1.9252 | 0.5671 | 0.5897 | 0.2905 | 1.0492 | ↓** | ↑ |
| 10 | Albafuran A | ESI- | C24H26O4 | 5.6517 | 423.1813247 | 0.815 | 0.0636 | 0.9952 | 0.02351 | 1.2943 | 1.3123 | ↓ | ↑* |
| 11 | Apigenin | ESI- | C15H10O5 | 6.7709 | 269.0461376 | 0.007359 | 0.9771 | 1.124 | 0.02572 | 1.2103 | 0.8 | ↑** | ↓* |
| 12 | Austalide L | ESI- | C25H32O6 | 6.2533 | 473.2194188 | 0.23 | 1.2634 | 4.3493 | 0.03318 | 2.1834 | 2.6437 | ↑ | ↑* |
| 13 | Cabreuvin | ESI- | C18H16O5 | 5.2546 | 357.0987859 | 0.04606 | 0.7263 | 0.9144 | 0.001463 | 1.1652 | 0.7803 | ↓* | ↓** |
| 14 | Cycloartomunin | ESI- | C26H24O7 | 6.0062 | 493.152403 | 0.5148 | 0.2849 | 0.9686 | 0.02696 | 1.3217 | 1.3115 | ↓ | ↑* |
| 15 | Cyclokievitone | ESI- | C20H18O6 | 13.8689 | 375.0880663 | 0.0009704 | 1.271 | 1.3495 | 0.0005049 | 1.9878 | 1.7047 | ↑*** | ↑*** |
| 16 | Epicatechin-(2beta->7,4beta->8)-epicatechin-(4beta->8)-epicatechin | ESI- | C45H36O18 | 3.4659 | 863.1852476 | 6.118E-5 | 1.7834 | 0.6623 | 0.3783 | 0.2338 | 1.0279 | ↓*** | ↑ |
| 17 | Eriodictyol | ESI- | C15H12O6 | 5.6297 | 287.0565516 | 0.02416 | 0.8703 | 0.882 | 0.01114 | 1.0476 | 0.7996 | ↓* | ↓* |
| 18 | Glicoisoflavanone | ESI+ | C22H24O6 | 4.2232 | 402.1918396 | 0.1352 | 0.8065 | 0.8698 | 0.003131 | 1.7776 | 1.6064 | ↓ | ↑** |
| 19 | Glucoliquiritin apioside | ESI- | C32H40O18 | 4.1121 | 693.2055156 | 0.00463 | 1.0554 | 0.8882 | 0.009555 | 1.2836 | 0.7888 | ↓** | ↓** |
| 20 | Glycyrrhizaflavonol A | ESI- | C20H18O7 | 4.0241 | 407.056393 | 0.03579 | 1.0072 | 1.1385 | 0.7412 | 0.1045 | 1.0064 | ↑* | ↑ |
| 21 | Heteroflavanone C | ESI- | C23H26O7 | 12.1909 | 413.1646242 | 0.6822 | 0.1799 | 1.0184 | 0.00198 | 1.5864 | 0.6626 | ↑ | ↓** |
| 22 | Heterophyllin | ESI- | C30H32O7 | 12.3494 | 549.2176164 | 1.539E-5 | 1.8727 | 1.5232 | 0.003511 | 1.7388 | 0.6539 | ↑*** | ↓** |
| 23 | Homopisatin | ESI- | C17H16O5 | 5.0967 | 345.0999454 | 6.508E-5 | 1.1876 | 1.1997 | 0.001912 | 0.8187 | 1.0892 | ↑*** | ↑** |
| 24 | Isoscoparin | ESI- | C22H22O11 | 4.179 | 461.1101987 | 0.003455 | 1.0059 | 0.8929 | 0.7811 | 0.1695 | 0.9801 | ↓** | ↓ |
| 25 | Isovitexin 2''-(6'''-p-coumaroylglucoside) | ESI+ | C36H36O17 | 4.3674 | 741.20051 | 0.03485 | 0.5189 | 0.9697 | 0.01993 | 1.2781 | 0.7959 | ↓* | ↓* |
| 26 | Kaempferol | ESI- | C15H10O6 | 6.9967 | 285.0410283 | 0.1017 | 0.9212 | 1.1812 | 0.01321 | 1.4654 | 0.6626 | ↑ | ↓* |
| 27 | Kaempferol 3-[6'''-p-coumarylglucosyl-(1->2)-rhamnoside] | ESI- | C36H36O17 | 4.333 | 739.1902097 | 0.611 | 0.1449 | 0.992 | 0.01408 | 1.4829 | 0.721 | ↓ | ↓* |
| 28 | Kanzonol M | ESI- | C23H26O6 | 13.6916 | 397.1692735 | 0.00205 | 1.7279 | 1.4592 | 0.04109 | 1.0226 | 0.8681 | ↑** | ↓* |
| 29 | Kanzonol N | ESI- | C22H24O6 | 8.4254 | 429.1592527 | 0.7121 | 0.1004 | 1.0056 | 1.277E-5 | 1.8551 | 0.5979 | ↑ | ↓*** |
| 30 | Kanzonol Z | ESI- | C25H26O5 | 14.2187 | 405.1744805 | 0.1143 | 0.5104 | 0.9651 | 0.000405 | 1.5445 | 0.7287 | ↓ | ↓*** |
| 31 | KB 2 | ESI+ | C25H26O8 | 4.513 | 437.1619976 | 0.6531 | 0.2499 | 0.9492 | 0.0001195 | 2.3697 | 2.24 | ↓ | ↑*** |
| 32 | Lampranthin II | ESI+ | C27H30O16 | 3.1413 | 611.1592442 | 0.09955 | 0.6079 | 0.9527 | 0.02745 | 1.2545 | 0.7934 | ↓ | ↓* |
| 33 | Licoricidin | ESI- | C26H32O5 | 5.4766 | 469.2241986 | 0.8962 | 0.1389 | 0.9217 | 0.0155 | 2.483 | 3.1377 | ↓ | ↑* |
| 34 | Loquatoside | ESI+ | C20H22O11 | 5.7317 | 403.1011849 | 0.9563 | 0.0392 | 1.0156 | 0.0129 | 2.107 | 2.1747 | ↑ | ↑* |
| 35 | Luteolin | ESI- | C15H10O6 | 5.7165 | 285.0410228 | 0.7278 | 0.1174 | 1.0079 | 0.002294 | 1.4459 | 0.7251 | ↑ | ↓** |
| 36 | Luteolin 4'-sulfate | ESI- | C15H10O9S | 4.559 | 364.9980091 | 0.02292 | 1.1368 | 0.8163 | 0.986 | 0.0635 | 0.9984 | ↓* | ↓ |
| 37 | Malvidin 3-(6''-acetyl-galactoside) | ESI- | C25H27O13+ | 10.45 | 570.1199123 | 0.08519 | 0.4403 | 1.0246 | 0.0007148 | 1.4727 | 0.7688 | ↑ | ↓*** |
| 38 | Mammeisin | ESI- | C25H26O5 | 6.1183 | 451.1777874 | 0.3715 | 0.706 | 44.3355 | 0.001657 | 2.8196 | 8.9252 | ↑ | ↑** |
| 39 | Morusinol | ESI+ | C25H26O7 | 4.4706 | 480.2040745 | 0.06637 | 1.8338 | 2.6916 | 0.0001941 | 2.3641 | 1.9104 | ↑ | ↑*** |
| 40 | Mulberrin | ESI- | C25H26O6 | 14.0448 | 403.1588388 | 0.5044 | 0.2014 | 0.9892 | 6.677E-5 | 1.5959 | 0.7308 | ↓ | ↓*** |
| 41 | Myricetin | ESI- | C15H10O8 | 5.761 | 299.0202237 | 4.899E-8 | 1.7562 | 0.5795 | 0.01225 | 0.4954 | 1.0732 | ↓*** | ↑* |
| 42 | Naringenin | ESI- | C15H12O5 | 6.7709 | 271.0617843 | 0.7001 | 0.1669 | 0.9823 | 0.00262 | 1.5362 | 0.6631 | ↓ | ↓** |
| 43 | Neocarlinoside | ESI- | C26H28O15 | 3.4659 | 579.1371044 | 0.001877 | 0.7433 | 0.9547 | 0.02765 | 1.1211 | 0.8636 | ↓** | ↓* |
| 44 | Neohesperidin dihydrochalcone | ESI- | C28H36O15 | 4.0684 | 633.1842059 | 0.01924 | 1.0365 | 0.8719 | 0.04005 | 1.2001 | 0.7764 | ↓* | ↓* |
| 45 | Naringin | ESI- | C21H22O8 | 14.1103 | 437.1038872 | 0.1811 | 0.7203 | 1.1714 | 0.001744 | 2.377 | 2.1395 | ↑ | ↑** |
| 46 | Orientin | ESI- | C21H20O11 | 4.0017 | 447.0944804 | 0.002747 | 1.166 | 0.8569 | 0.5831 | 0.2296 | 1.0261 | ↓** | ↑ |
| 47 | Phaseollidin | ESI+ | C20H20O4 | 9.7552 | 357.1684388 | 0.006969 | 1.4379 | 0.7399 | 0.2914 | 0.5266 | 0.9238 | ↓** | ↓ |
| 48 | Phaseollinisoflavan | ESI+ | C20H20O4 | 6.8511 | 366.1691282 | 0.005571 | 1.7077 | 0.7028 | 0.05814 | 1.0168 | 0.8031 | ↓** | ↓ |
| 49 | Pinocembrin | ESI- | C15H12O4 | 10.2485 | 255.066514 | 0.2996 | 0.3941 | 0.9604 | 0.03929 | 1.2836 | 0.7125 | ↓ | ↓* |
| 50 | Quercetin | ESI- | C15H10O7 | 5.761 | 301.0359049 | 2.528E-8 | 1.2355 | 0.8027 | 6.951E-6 | 1.1425 | 0.7645 | ↓*** | ↓*** |
| 51 | Quercetin 3-glucoside 7-xyloside | ESI- | C26H28O16 | 3.4876 | 577.1213906 | 0.002493 | 0.725 | 0.9469 | 0.01908 | 1.0399 | 0.8577 | ↓** | ↓* |
| 52 | Rubraflavone B | ESI+ | C30H34O5 | 2.752 | 516.2790599 | 0.002208 | 1.023 | 1.0997 | 0.004464 | 0.984 | 1.0927 | ↑** | ↑** |
| 53 | Rubraflavone C | ESI+ | C30H34O6 | 7.7373 | 508.2712244 | 0.0366 | 1.6715 | 1.584 | 0.3923 | 0.6508 | 1.1143 | ↑* | ↑ |
| 54 | Sativan | ESI+ | C17H18O4 | 6.9155 | 269.1165045 | 1.265E-5 | 1.4899 | 1.2894 | 0.08397 | 0.3764 | 0.9786 | ↑*** | ↓ |
| 55 | Swertiajaponin | ESI+ | C22H22O11 | 4.3037 | 463.1224626 | 0.001889 | 1.1113 | 0.879 | 0.2303 | 0.5607 | 0.9358 | ↓** | ↓ |
| 56 | Taxifolin 3-arabinoside | ESI- | C20H20O11 | 6.2082 | 417.0836238 | 0.000416 | 1.0017 | 0.8921 | 3.521E-5 | 1.4741 | 0.7133 | ↓*** | ↓*** |
| 57 | Tectoridin | ESI+ | C22H22O11 | 4.7275 | 463.1223793 | 6.731E-5 | 2.0446 | 0.5722 | 0.4311 | 0.3299 | 1.0659 | ↓*** | ↑ |
| 58 | Tricin | ESI- | C17H14O7 | 6.9967 | 329.0673464 | 0.01206 | 1.5025 | 1.4361 | 0.00795 | 1.3855 | 0.7216 | ↑* | ↓** |
| 59 | Viniferal | ESI- | C35H26O8 | 10.0022 | 609.1315125 | 0.009819 | 0.616 | 0.9604 | 0.02688 | 1.4858 | 0.7136 | ↓** | ↓* |
| 60 | Vitisin B | ESI+ | C25H25O12+ | 3.4822 | 581.1490822 | 0.004805 | 0.8015 | 0.9478 | 0.01496 | 1.1582 | 0.8639 | ↓** | ↓* |

ESI, electrospray ionization; RT, retention time; VIP, variable importance in the projection. **p*<0.05, ***p*<0.01, ****p*<0.001.

**Supplementary Table 2.** Number of microbial ASVs per sample in HFDYM across three fermentation periods.

|  | Bacteria | Fungi |
| --- | --- | --- |
| A0_1 | 38 | 26 |
| A0_2 | 73 | 104 |
| A0_3 | 84 | 110 |
| B45_1 | 215 | 30 |
| B45_2 | 169 | 24 |
| B45_3 | 228 | 23 |
| B90_1 | 81 | 13 |
| B90_2 | 116 | 133 |
| B90_3 | 159 | 59 |

**Supplementary Table 3.** Enzymes Involved in the Transformation Pathways of Ten Flavonoids in HFDYM by Strains A-3 and B-7.

| EC number or abbreviation | Full name of enzyme |
| --- | --- |
| 1.1.1.219 | Bifunctional dihydroflavonol 4-reductase/flavanone 4-reductase |
| 1.14.11.9 | Naringenin 3-dioxygenase |
| 1.14.14.162 | Flavanone 2-hydroxylase |
| 1.14.14.81 | Flavonoid 3',5'-hydroxylase |
| 1.14.14.82 | Flavonoid 3'-monooxygenase |
| 1.14.19.76 | Flavone synthase II |
| 1.14.20.5 | Flavone synthase I |
| 1.14.20.6 | Flavonol synthase |
| 1.17.1.3 | Leucoanthocyanidin reductase |
| 2.4.1.185 | Flavanone 7-O-beta-glucosyltransferase |
| 2.4.1.236 | Flavanone 7-O-glucoside 2''-O-beta-L-rhamnosyltransferase |
| TcCGT1 | C-glycosyltransferase from *Trollius chinensis* |

**Supplementary Methods 1**

**Sample Preparation for HPLC Analysis of Ten Flavonoids**

Preparation of test solutions

The test solutions were prepared according to the following procedure. Approximately 18 g of each sample (from groups M, M+A-3, M+B-7, W, W+A-3, and W+B-7) was accurately weighed and transferred into a conical flask. Then, 200 mL of 60% methanol was precisely added. The mixture was subjected to ultrasonic extraction for 40 minutes and subsequently filtered. The filtrate was collected in an eggplant-shaped flask and concentrated to dryness under reduced pressure. The residue was reconstituted in 10 mL of purified water and then extracted with ethyl acetate repeatedly until the ethyl acetate layer became colorless. The combined ethyl acetate layers were transferred to a new eggplant-shaped flask and evaporated to dryness under reduced pressure. The final residue was dissolved in methanol, quantitatively transferred to a 1 mL volumetric flask, and diluted to volume with methanol. Prior to HPLC injection, the solution was filtered through a 0.22 μm microporous membrane.

Preparation of standard solution

A mixed standard stock solution was prepared by accurately weighing the following reference compounds: catechin, vitexin, naringin, myricetin, eriodictyol, luteolin, quercetin, naringenin, apigenin, and kaempferol. Each compound was dissolved in methanol to prepare a solution with the specified concentration: 0.172, 0.180, 0.184, 0.188, 0.176, 0.128, 0.184, 0.164, 0.180, and 0.192 mg/mL, respectively.
